# Dorsal raphe nucleus–hippocampus serotonergic circuit underlies the depressive and cognitive impairments in 5×FAD male mice

**DOI:** 10.1186/s40035-024-00425-w

**Published:** 2024-07-24

**Authors:** Meiqin Chen, Chenlu Wang, Yinan Lin, Yanbing Chen, Wenting Xie, Xiaoting Huang, Fan Zhang, Congrui Fu, Kai Zhuang, Tingting Zou, Dan Can, Huifang Li, Shengxi Wu, Ceng Luo, Jie Zhang

**Affiliations:** 1 The Key Laboratory of Neural and Vascular Biology, Ministry of Education, College of Basic Medicine, Hebei Medical University, Shijiazhuang, 050017 China; 2 Institute of Neuroscience, College of Medicine, Xiamen University, Xiamen, 361102 China; 3 Department of Anesthesiology, First Affiliated Hospital of Xiamen University, Xiamen, 361000 China; 4 Department of Neurobiology, School of Basic Medicine, Fourth Military Medical University, Xi’an, 710032 China; 5 Department of Neurology, Sichuan Provincial People’s Hospital, School of Medicine, University of Electronic Science and Technology of China, Chengdu, 610054 China; 6 Institute of Neuroscience, Fujian Medical University, Fuzhou, 350004 China

**Keywords:** Alzheimer’s disease, Depressive symptoms, Cognitive impairment, 5-HT neurons, Dorsal raphe nucleus, Dorsal hippocampal CA1, Serotonin receptor

## Abstract

**Background:**

Depressive symptoms often occur in patients with Alzheimer's disease (AD) and exacerbate the pathogenesis of AD. However, the neural circuit mechanisms underlying the AD-associated depression remain unclear. The serotonergic system plays crucial roles in both AD and depression.

**Methods:**

We used a combination of in vivo trans-synaptic circuit-dissecting anatomical approaches, chemogenetic manipulations, optogenetic manipulations, pharmacological methods, behavioral testing, and electrophysiological recording to investigate dorsal raphe nucleus serotonergic circuit in AD-associated depression in AD mouse model.

**Results:**

We found that the activity of dorsal raphe nucleus serotonin neurons (DRN^5-HT^) and their projections to the dorsal hippocampal CA1 (dCA1) terminals (DRN^5-HT^-dCA1^CaMKII^) both decreased in brains of early 5×FAD mice. Chemogenetic or optogenetic activation of the DRN^5-HT^-dCA1^CaMKII^ neural circuit attenuated the depressive symptoms and cognitive impairments in 5×FAD mice through serotonin receptor 1B (5-HT_1B_R) and 4 (5-HT_4_R). Pharmacological activation of 5-HT_1B_R or 5-HT_4_R attenuated the depressive symptoms and cognitive impairments in 5×FAD mice by regulating the DRN^5-HT^-dCA1^CaMKII^ neural circuit to improve synaptic plasticity.

**Conclusions:**

These findings provide a new mechanistic connection between depression and AD and provide potential pharmaceutical prevention targets for AD.

**Supplementary Information:**

The online version contains supplementary material available at 10.1186/s40035-024-00425-w.

## Introduction

Alzheimer's disease (AD) is one of the major neurodegenerative diseases characterized by irreversible and progressive memory loss. In addition to cognitive impairments, neuropsychiatric symptoms (NPS) are often found in AD patients. One major NPS associated with AD is depression [1]. Depressive symptoms emerge in the early stage of AD [2]. Meanwhile, cognitive dysfunction is also a distinctive feature of major depressive disorder. The main pathological features of AD are extracellular amyloid β (Aβ) plaques and intracellular neurofibrillary tangles (NFT) [3]. The Aβ deposition and NFT induce synaptic dysfunction and neurodegeneration, which will disrupt the neural circuits associated with cognition and emotion [4–7]. However, the underlying mechanisms of AD-associated depression, especially the underlying neural circuits, remain unclear.

Dysfunction of the serotonergic system [8–11] and loss of serotonin (or 5-hydroxytryptamine, 5-HT) neurons [12] are closely associated with cognitive deficits in AD patients. Selective serotonin reuptake inhibitors can effectively improve AD-related cognitive function and emotional behavior [13]. In the central nervous system (CNS), serotonergic neurons are mostly located in two brain regions: the dorsal raphe nucleus (DRN) and the medial raphe nucleus (MRN) [14], and participate in many biological processes, including sleep-wake cycles, emesis, appetite, mood, memory, etc. The serotonergic system contains 14 receptor subtypes, of which 13 are G protein-coupled receptors (GPCRs), and one is a ligand-gated cation channel [15]. Among these serotonin receptors, several are closely related with depression and AD. The 5-HT_1B_ receptor (5-HT_1B_R), a putative target in the pharmacologic treatment of depression [16], is the inhibitory Gi-coupled protein receptor that mediates the adenylate cyclase activity [17]. The 5-HT_4_ receptor (5-HT_4_R) is highly expressed in the hippocampus (HPC), amygdala, prefrontal cortex, and striatum [18], and participates in the AD pathogenesis [19]. Serving as an excitatory Gs-coupled protein receptor, 5-HT_4_R is capable of  promoting the excitability of neurons [18, 20].

The hippocampal CA1 region is associated with spatial learning and memory, and is one of the most vulnerable brain regions in AD [21]. Hippocampal pyramidal neurons of CA1 highly express serotonin receptors, such as 5-HT_1B_R and 5-HT_4_R [22]. 5-HT_1B_R regulates the release of glutamate, and its activation suppresses synaptic transmission in terminals of CA1 pyramidal cells [23]. The activation of axonal terminals of 5-HT neurons in the hippocampal CA1 enhances the excitatory transmission of CA3-CA1 synapses and improves spatial memory [24]. As another important serotonin receptor, the 5-HT_4_R level has been found decreased in the HPC and prefrontal cortex of AD patients [25]. Accumulating evidence reports that 5-HT_4_R agonists could increase hippocampal dendritic spine density [26] and improve learning and memory [24], while their antagonists impair associative memory [27]. However, the implications of DRN^5-HT^ neurons and hippocampal serotonin receptors in AD are unclear.

In this study, we found that the early 5×FAD mice (3 months old) exhibited depressive-like behaviors, and decreased 5-HT levels in DRN and dorsal hippocampal CA1 (dCA1) compared with wild-type (WT) mice. We used a combination of in vivo trans-synaptic circuit-dissecting anatomical approaches, chemogenetic manipulations, optogenetic manipulations, pharmacological methods, behavioral testing, and electrophysiological recording to reveal that the DRN–HPC  serotonergic circuit may be a new pathway for AD-associated depression, and that targeting 5-HT_1B_R/5-HT_4_R is a potential therapeutic intervention for AD.

## Materials and methods

### Animals

5×FAD mice (B6SJL-Tg (APPSwFlLon, PSEN1*M146L*L286V) 6799Vas/Mmjax Strain #034840-JAX), CaMKII-Cre and GAD2-Cre male mice were obtained from Jackson laboratory (Bar Harbor, ME). Experimental and control mice were generated by crossing male 5×FAD heterozygote mice to C57BL/6 J females. Adult WT male mice and 5×FAD male mice were housed in a 12-h light/dark interval with ad libitum access to food and water and housed at 22 ± 2 °C, except during behavioral tests. All mice were housed at the Laboratory Animal Center at Xiamen University. All experimental procedures were performed according to protocols approved by the Institutional Animal Care and Use Committee at Xiamen University, and the protocols were performed under strict adherence to the Animal Research: Reporting of in vivo Experiments (ARRIVE) guidelines.

### Stereotaxic surgery and virus injections

Before surgery, the mice were anesthetized with an intraperitoneal injection of pentobarbital (20 mg/kg) and placed on a stereotactic frame (RWD Life Science, Shenzhen, China). Viral vectors were injected into the DRN (coordinates: AP, −4.62 mm from Bregma; ML, 0 mm; DV, −3.4 mm from the brain surface) and the dCA1 bilaterally (AP, −1.8 mm from Bregma; ML, ± 1.5 mm; DV, −1.5 mm from the brain surface) at a slow rate of 30 nl/min.

For analysis of whole-brain projections of DRN^5-HT^ neurons, AAV-EF1α-Dio-mCherry (AAV2/9, titer: 2.88 × 10^12^ vg/ml, 200 nl, Brain VTA Technology, Wuhan, China) and AAV-TPH2-Cre (AAV2/9, 5.13 × 10^12^ vg/ml, 200 nl, Brain VTA Technology) at a 1:1 ratio was injected into the DRN of mice.

For retrograde tracing, 200 nl of cholera toxin-B (CTB) (Brain VTA Technology) was stereotactically injected into the dCA1 at a flow rate of 20 nl/min.

For retrograde tracking, the AAV-TPH2-Cre (AAV2/R, titer: 5.81 × 10^12^ vg/ml, 200 nl, Brain VTA Technology) was injected into the dCA1, and the Cre-dependent virus AAV-EF1α-Dio-hM3Dq (Gq)-mCherry (AAV2/9, titer: 2.77 × 10^12^ vg/ml, 500 nl, Brain VTA Technology) was injected into the DRN. After 3 weeks, mice were anesthetized with an intraperitoneal injection of pentobarbital (20 mg/kg) and transcardially perfused. Brain slices were prepared for tracing the mCherry signal or co-staining with tryptophan hydroxylase 2 (TPH2)-specific antibodies in the DRN.

For monosynaptic anterograde tracing, AAV-TPH2-Cre (AAV2/1, 1.02 × 10^13^ vg/ml, 300 nl, Brain VTA Technology) was injected into the DRN of C57 mice to allow its anterograde spreading to the downstream soma to express *Cre *[28]. Simultaneously, AAV2/9-Ef1α-Dio-hM3Dq (Gq)-mCherry (AAV2/9, titer: 2.77 × 10^12^ vg/ml, 250 nl, Brain VTA Technology) was injected into the dCA1. After 3 weeks, mice were anesthetized with an intraperitoneal injection of pentobarbital (20 mg/kg) and transcardially perfused. Brain slices were prepared for tracing the mCherry signal or co-staining with CaMKIIα-specific antibodies or GAD67-specific antibodies in the dCA1.

For retrograde monosynaptic tracing, helper viruses that contain AAV-Ef1α-Dio-oRVG-WPRE-pA (AAV-Ef1α-Dio–oRVG, AAV2/9, 5.22 × 10^12^ vg/ml, 100 nl, Brain VTA Technology) and AAV-Ef1α-Dio-His-EGFP-2a-TVA-WPRE-pA (AAV-Ef1α-Dio-TVA–EGFP, AAV2/9, 5.53 × 10^12^ vg/ml, 100 nl, Brain VTA Technology) at a 1:1 ratio was injected into the dCA1 of CaMKII-Cre mice or GAD2-Cre mice. Three weeks later, RV-EnVA-ΔG–DsRed (3.10 × 10^8^ IFU/ml, 250 nl, Brain VTA Technology) was injected into the dCA1 using the identical conditions and coordinates. The modified rabies virus cannot infect neurons and spread retrogradely without the TVA and rabies glycoprotein component, which is provided by AAV helpers.

For chemogenetic manipulation, the AAV-TPH2-Cre (AAV2/R, titer: 5.81 × 10^12^ vg/ml, 200 nl, Brain VTA Technology) was injected into the dCA1, and the Cre-dependent virus AAV-EF1α-Dio-mCherry (AAV2/9, titer: 2.88 × 10^12^ vg/ml, 500 nl, Brain VTA Technology) or AAV-EF1α-Dio-hM3Dq (Gq)-mCherry (AAV2/9, titer: 2.77 × 10^12^ vg/ml, 500 nl, Brain VTA Technology) was injected into the DRN.

For optogenetic manipulation, the AAV-TPH2-Cre (AAV2/R, titer: 5.81 × 10^12^ vg/ml, 200 nl, Brain VTA Technology) was injected into the dCA1. AAV-EF1α-Dio-mCherry (AAV2/9, titer: 2.88 × 10^12^ vg/ml, 500 nl, Brain VTA Technology) or AAV-EF1α-Dio-hChR2(H134R)-mCherry (AAV2/9, titer: 4.83 × 10^12^ vg/ml, 500 nl, Brain VTA Technology) was injected into the DRN of mice. The single monofiber implant (diameter 200 μm, Numerical Aperture (NA): 0.37, RWD Life Science, Shenzhen, China) was positioned above the injection sites. The implant was secured to the skull with dental cement. Mice were allowed to recover for at least 3 weeks before behavioral experiments. The delivery of a 2-min pulse of blue light (473 nm, 4–5 mW, 20 Hz) was controlled using a Master-8 pulse stimulator (RWD). An identical stimulus protocol was applied to the control mice. The location of the fiber implants was examined in all mice after the experiments, and mice in which the fiber implants were located outside the desired brain region were excluded from analysis.

For chemogenetic manipulation, an intraperitoneal injection of Clozapine N-oxide (CNO, 3 mg/kg, APEXBIO, Houston, TX, Cat# A3317) was given 30 min before the behavior tests.

For TPH2 overexpression in DRN^5-HT^ neurons, AAV-TPH2-mCherry (AAV2/9, titer: 5.62 × 10^12^ vg/ml, 1 μl, Brain VTA Technology) or AVV-TPH2-Tph2-mCherry (AAV2/9, titer: 5.60 × 10^12^ vg/ml, 1 μl, Brain VTA Technology) was injected into the DRN of mice. The viruses are listed in Additional file 1: Table S1.

### Intraperitoneal injection and cannula infusion of drugs

CNO (APEXBIO, Cat# A3317) was delivered via intraperitoneal injection at a concentration of 3 mg/kg 30 min before behavioral experiments. For cannula microinjection experiments, an internal stainless-steel injector attached to a 10-μl syringe (Hamilton) and a microinfusion pump (RWD) was inserted into the guide cannula (internal diameter 0.34 mm, RWD) and used to infuse CP93129 (1.5 μg/µl, 1 µl, Tocris Bioscience, Shanghai, China, Cat# 1032) into the bilateral dCA1 at a flow rate of 200 nl/min [29]. Similarly, BIMU8 (2 μg/µl, 1 µl, Tocris, Cat# 4397) was locally applied [29]. Control mice were given the same volume of saline.

### Behavioral studies

As described above, the virus was injected into the DRN or the dCA1 of 2-month-old mice by stereotactic microinjection. Depressive behavior tests were performed at 3 months of age and cognitive behavior tests were detected at 6 months of age. All mice of chemogenetic manipulation were intraperitoneally injected with CNO (3 mg/kg, APEXBIO, A3317) 30 min before behavioral testing. To examine whether 5-HT_1B_R and 5-HT_4_R are involved in the effects of activation of DRN^5-HT^ neurons on mouse behavior, agonist for 5-HT_1B_R (CP93129, 1 0.5 μg/µl, 1 µl, dCA1 locational injection, Tocris, Cat# 1032) or 5-HT_4_R (BIMU8, 2 μg/µl, 1 µl, dCA1 locational injection, Tocris, Cat# 4397) was administered once daily for 7 days prior to the behavioral test.

### Tail-suspension test (TST)

A mouse was suspended approximately 50 cm above the table, with adhesive tape placed about 1 cm from the tip of the tail. The mouse behavior was monitored from the side with a video tracking system for 6 min (2 min for adaptation and 4 min for recording). The immobility time was recorded.

### Forced swimming test (FST)

Mice were individually placed in a hyaline cylinder (diameter 15 cm, height 25 cm) filled with fresh water (with temperature maintained at 22–25 °C) to a depth of 15 cm. Each mouse was forced to swim for 6 min. The immobility time during the last 4 min was manually counted and analyzed by an observer who was blind to the treatment conditions.

### Sucrose preference test (SPT)

The SPT consists of two phases, the adaption and the testing phases. During the adaption phase, a mouse was acclimatized to two bottles of 1% sucrose solution (*w*/*v*) in each cage. After 24 h, the bottles were changed to water. To avoid side bias, the positions of the two bottles were switched after 12 h. After adaptation, the mice were deprived of water and food for 24 h. Then the mice were housed individually in the cages with free access to two bottles, one containing water and another containing 1% sucrose solution for 12 h. Th volumes of consumed sucrose solution and water were recorded, and sucrose preference (SP) was calculated as: SP (%) = sucrose consumption / (sucrose consumption + water consumption) × 100%.

### Morris water maze (MWM)

A tank of 120 cm in diameter was filled with water and made opaque with a white, non-toxic titanium dioxide maintained at 22 ± 2 °C. The maze was labeled with several distinct extra-maze cues. A camera fixed at a distance of 1 m from the water surface was connected to a digital tracking device. The tracking information was then processed by a computer with the MWM software. Thereafter, escape latency was recorded in the training phase. After the training stage, the platform was removed. The times of crossing the platform, the latency to reach the target platform, and the time spent in each quadrant were recorded during the testing phase.

### Open field test (OFT)

A mouse was placed in a square reaction chamber (40 × 40 cm^2^ chamber) and allowed to explore freely for 10 min. A camera was used to record the free movement of the mouse. The time spent in the central and the peripheral areas and the total distance traveled were analyzed to quantify anxiety.

### Elevated plus maze (EPM)

A mouse was put in a plus-shaped maze and allowed to explore freely for 5 min. The maze was cross-shaped, consisting of two open arms and two closed arms. The connecting part is the central area, at a certain height from the ground. A camera was used to record the free movement of the mouse. Anxiety was quantified by analyzing the percentages of time spent in the central, open, and closed areas.

### Light-dark transition test (LDT)

A mouse was placed in a light-dark response chamber and allowed to explore freely for 5 min. The experimental facility consisted of a dark safety compartment and a bright aversion compartment. A camera was used to record the free movement of the mouse. The number of switches between the compartments was analyzed to quantify anxiety.

### Synaptic plasticity/long-term potentiation (LTP)

Neurons in slices were visualized using a 40× water immersion objective on an upright microscope (BX51WI, Olympus) equipped with infrared-differential interference contrast (IR-DIC) and an infrared camera connected to a video monitor. Whole-cell patch-clamp recordings were obtained from visually identified DRN and HPC cells. Patch pipettes (3–5 MΩ) were pulled from borosilicate glass capillaries (Borosilicate glass, Sutter Instruments, CA) with an outer diameter of 1.5 mm on a four-stage horizontal puller (P1000, Sutter Instruments, CA). Signals were acquired via a Multiclamp 700B amplifier, low-pass filtered at 2.8 kHz, digitized at 10 kHz, and analyzed with Clampfit 10.7 software (Molecular Devices, Forster City, CA). The experimental recording was immediately terminated if the series resistance changed more than 20% during the recording.

We used extracellular field potential recording to investigate LTP changes in the hippocampal CA1 region. Mice of desired genotypes were anesthetized with 3%–5% isoflurane. Transverse hippocampal slices (400 μm thickness) were prepared using a Vibroslice (VT 1000S; Leica) in an ice-cold solution containing 64 mM NaCl, 2.5 mM KCl, 1.25 mM NaH_2_PO_4_, 10 mM MgSO_4_, 0.5 mM CaCl_2_, 26 mM NaHCO_3_, 10 mM glucose, and 120 mM sucrose. Slices were allowed to recover for 30 min at 32 °C and then at room temperature (25 ± 1 °C) for at least 1 h prior to initiation of recording in the artificial cerebrospinal fluid (ACSF) (1.3 mM Mg^2+^) containing 120 mM NaCl, 3.5 mM KCl, 1.25 mM NaH_2_PO_4_, 1.3 mM MgSO_4_, 2.5 mM CaCl_2_, 26 mM NaHCO_3_, and 10 mM glucose. Slices were transferred to the recording chamber and perfused with ACSF (3 ml/min) at 32 °C. Brain slices were transferred to an interface chamber to facilitate long-term slice viability, and superfused with ACSF (1.3 mM Mg^2+^) saturated with 95% O_2_/5% CO_2_. Field excitatory postsynaptic potentials (fEPSPs) were recorded using a glass patch electrode in the CA1 stratum radiatum layer in response to Schaffer collateral stimulation. The patch electrode had an electrical resistance of 1–2 MΩ at 1 kHz when filled with ACSF (1.3 mM Mg^2+^). The Schaffer collateral inputs to the CA1 region were stimulated with a bipolar tungsten electrode. Field excitatory postsynaptic potentials (fEPSPs) were recorded from neurons within the CA1 dendritic layer by inserting an electrode in the Schaffer collateral pathway as the stimulating electrode.

For fEPSP recordings from the HPC, a stimulus-response (input–output) curve was first obtained by measuring the fEPSP slope (first 1-ms response after fiber volley) as a function of the fiber volley amplitude, which was used to quantify basal synaptic transmission strength. We then chose a stimulus intensity that produced a 30% maximum fEPSP amplitude throughout the experiments. fEPSPs evoked by this stimulus intensity were recorded for 20 min to establish a baseline.

After the 20-min baseline responses of stimulus-induced fEPSPs, CNO (5 μM), CP93129 (5-HT_1B_R agonist, 5 μM, Tocris Bioscience, Cat# 1032), NAS-181 (5-HT_1B_R antagonist, 5 μM, MedChemExpress, Cat# HY-103156), or BIMU8 (5-HT_4_R agonist, 5 μM; Tocris Bioscience, Cat# 4397) was perfused at 3 ml/min and recorded for 30 min [29]. Then an LTP induction stimulation protocol was applied. To elicit LTP, we used a theta burst stimulation protocol, which consisted of a 2-s-long 5-Hz train (each train consisting of four pulses at 100 Hz) repeated 5 times at 10-s intervals. Following LTP induction, fEPSP responses were recorded for an additional 1 h.

### Whole-cell patch-clamp recording

Neurons in the slices were visualized using a 40× water immersion objective on an upright microscope (BX51WI, Olympus) equipped with infrared-differential interference contrast (IR-DIC) and an infrared camera connected to a video monitor. Whole-cell patch-clamp recordings were obtained from visually identified DRN and HPC cells. Patch pipettes (3–5 MΩ) were pulled from borosilicate glass capillaries (VitalSense Scientific Instruments Co., Ltd) with an outer diameter of 1.5 mm on a four-stage horizontal puller (P1000, Sutter Instruments). The signals were acquired via a Multiclamp 700B amplifier (Molecular Devices), low-pass filtered at 2.8 kHz, digitized at 10 kHz, and analyzed with the Clampfit 10.7 software (Molecular Devices). If the series resistance changed more than 20% during the recording, the experimental recording was immediately terminated.

Spontaneous excitatory postsynaptic currents (sEPSCs) were recorded at a holding potential of − 70 mV. For sEPSCs recording, glass pipettes were filled with the solution containing 140 mM CsCH_3_SO_3_, 5 mM TEA-CI, 10 mM Hepes, 1 mM EGTA, 2 mM MgCl_2_, 2.5 mM Mg-ATP, and 0.3 mM Na-GTP (pH 7.3, osmolarity of 285–290 mOsm/kg). For spontaneous inhibitory postsynaptic currents (sIPSCs) recording, the same pipette solution was used.

The pipettes were filled with an intracellular solution that contained (in mM) 125 K-gluconate, 10 HEPES, 2 MgCI_2_, 0.3 GTP-Na_2_, 5 NaCl, and 10 EGTA (pH 7.2, osmolarity of 285–290 mOsm/kg). To assess the intrinsic excitability of neurons in the DRN and dCA1 regions, we injected 1-s current steps (0 pA to 120 pA, with 10 pA increment) at −70 mV holding potential. Data were filtered at 2 kHz and sampled at 10 kHz. Neurons with a resting potential of at least −70 mV and resistance that fluctuated within 15% of initial values (< 20 MΩ) were analyzed. Mini-events were analyzed with the MiniAnalysis software.

### Nissl staining

Coronal frozen sections of HPC (30 μm thick) were washed with distilled water, and stained with Nissl staining solution (Beyotime, China) for 5 min at 37 ℃. Then, the sections were washed twice with distilled water for a few seconds, rinsed with 95% ethanol for 5 min, and air-dried. Sections were washed twice in xylene for 5 min each and sealed with neutral balsam.

### Immunohistochemistry

Mice were anesthetized with 4% isoflurane, followed by transcardial blood clearing with 0.01 M PBS and fixation with 4% ice-cold paraformaldehyde (PFA) in 0.1 M phosphate buffer (pH 7.4). For 0.01 M PBS, the composition consisted of 140 mM NaCl, 2 mM KCl, 10 mM Na_2_HPO_4_·12H_2_O and 2 mM KH_2_PO_4_. Brains were post-fixed in 4% PFA overnight at 4 °C and cryoprotected for 48 h in 30% sucrose. The brains were then embedded in OCT, frozen at −20 °C, and sectioned into 30-μm sections on a sliding microtome (Leica CM1950, Germany). Following extensive washes in 0.01 M PBS, the free-floating sections were blocked in primary antibody solution (5% normal goat serum, 0.3% TritonX-100, in 0.01 M PBS) for 0.5 h, and incubated with anti-TPH2 (1:1000, Rabbit, Novus, Littleton, CO, Cat# NB100-74555), anti-c-Fos (1:500, Rabbit, Cell Signaling Technology, Danvers, MA, Cat# 2250), or anti-CaMKIIα (1:500, Rabbit, Novus, Cat# NB100-1983) at 4 °C for 24 h. Sections were washed in 0.01 M PBS, incubated with secondary antibodies (Invitrogen, Carlsbad, CA, 1 μg/ml), and mounted on glass slides using DAPI-containing mounting medium. Images were acquired on an Olympus FV1000MPE-B confocal microscope (Tokyo, Japan) with a 20 × dry air or 60 × oil immersion objective. Image acquisition parameters (e.g., laser power, pinhole size, detector gain, and offset) were kept constant to enable signal intensity comparisons. The primary antibodies are listed in Additional file 1: Table S2.

### Enzyme linked immunosorbent assay (ELISA)

The contents of 5-HT in the dCA1 and the DRN were determined by ELISA (COIBO BIO, shanghai, China). The relevant reagents were prepared in strict accordance with the method indicated in the instructions. The standard wells and sample wells were set on the enzyme plate. The standard wells were added with 50 μl standard substance of different concentrations, and the sample wells were added with 50 μl sample (supernatant of the dCA1 and the DRN homogenate). Then 40 μl of sample diluent was added, followed by addition of 100 μl HRP-labeled detection antibody. The wells were sealed, incubated for 60 min at 37 °C, and patted dry on absorbent paper after discarding the liquid. The plate was washed for 5 times, added with 50 μl substrate A and B to each well, incubated for 15 min at 37 °C, and added with 50 μl termination solution to each well. The OD value of each well was measured at 450 nm to calculate the concentrations of 5-HT in the samples.

### Western blotting

The dCA1 or DRN tissue was immediately homogenized in ice-cold radioimmunoprecipitation assay buffer (Boster, Wuhan, China) containing a cocktail of protease inhibitors. The lysates were then centrifuged at 12,000 rpm for 10 min at 4 °C and supernatants were collected. The total protein concentration was measured using a bicinchoninic acid (BCA) protein kit (LifeTech, Shenzhen, China). Western blot was performed as described previously [30]. Briefly, equal amounts (50 μg) of proteins were separated by SDS-PAGE. After electrophoresis, the separated proteins were transferred onto PVDF (polyvinylidene difluoride) membranes in an ice-cold buffer (25 mM Tris HCl, 192 mM glycine, and 20% methanol) by electrotransfer for 1.5 h, and blocked in TBST containing 5% non-fat milk for 2 h at room temperature. The membranes were washed and incubated with primary antibody in 5% non-fat milk at 4 °C overnight: anti-APP (1:2000, Rabbit, Sangon Biotech, Shanghai, China, Cat# D260097-0025), anti-TPH2 (1:2000, Rabbit, Novus, Cat# NB100-74555), anti-GAPDH (1:5000, Mouse, Protientech, Chicago, IL, Cat# 6004-1-lg). On the next day, membranes were washed three times with TBST, followed by incubation with horseradish peroxidase-linked secondary antibodies for 1 h at room temperature. After another round of TBST wash, chemiluminescent Western ECL detection reagents (Bio-Rad, Hercules, CA, Cat# 1705061) were used to illuminate the signals. Integrated gray values of each band were quantified using the ImageJ software. The primary antibodies involved are listed in Table S2.

### Quantitative reverse transcription PCR (qRT-PCR)

The dCA1 tissue was microdissected as described above. Total RNA was extracted with Trizol™ Reagent according to the manufacturer’s instructions (Invitrogen). The concentration of total RNA was measured with a NanoDropTM OneC spectrophotometer (Thermo Fisher Scientific, Waltham, MA). The RT reaction was performed at 37 °C for 15 min, 50 °C for 5 min, 98 °C for 5 min, and 4 ℃ hold using a High-Capacity cDNA Reverse Transcription Kit with RNase Inhibitor (Toyobo, FSQ-201). qRT-PCR was then performed using the Step OnePlus™ Real-Time PCR System (Thermo Fisher Scientific). The 10 μl reaction mix was composed of 5 μl of PowerUpTM SYBRTM Green Master Mix (Roche), 1 μl of each primer, 2 μl of cDNA and 1 μl of ddH_2_O. All reactions were run on a Light Cycler 480 Instrument II (Roche Diagnostics, Basel, Switzerland) with a 15-min hot start at 95 °C followed by 40 cycles of a 3-step thermocycling program: denaturation at 94 °C for 15 s; annealing at 55 °C for 30 s; and extension at 70 °C for 30 s. Samples were assessed in triplicate in each experiment. The relative levels of target genes were analyzed by the 2^−ΔΔCt^ method, normalizing the cycle threshold (Ct) values of the cytokine receptor mRNAs to the Ct value of β-actin. Data were quantified as the fold change relative to WT mice. Primers are shown in Table 1.

**Table 1:** The primer sequences used for qPCR

Gene	Primer sequences (5′ −> 3′)
5-HT_1A_R	
Forward	GACAGGCGGCAACGATACT
Reverse	CCAAGGAGCCGATGAGATAGTT
5-HT_1B_R	
Forward	CGCCGACGGCTACATTTAC
Reverse	TAGCTTCCGGGTCCGATACA
5-HT_1D_R	
Forward	ATCACCGATGCCCTGGAGTA
Reverse	GCCAGAAGAGTGGAGGGATG
5-HT_1F_R	
Forward	ATCAACTCCCTCGTGATCGC
Reverse	ACACGTACAACAGATGATGTCG
5-HT_2A_R	
Forward	TAATGCAATTAGGTGACGACTCG
Reverse	GCAGGAGAGGTTGGTTCTGTTT
5-HT_2B_R	
Forward	GAACAAAGCACAACTTCTGAGC
Reverse	CCGCGAGTATCAGGAGAGC
5-HT_2C_R	
Forward	CTAATTGGCCTATTGGTTTGGCA
Reverse	CGGGAATTGAAACAAGCGTCC
5-HT_3A_R	
Forward	CCTGGCTAACTACAAGAAGGGG
Reverse	TGCAGAAACTCATCAGTCCAGTA
5-HT_3B_R	
Forward	CTGTCTACCTGGACCTTTGCG
Reverse	AACTCATCGTTCCAAACCTCTC
5-HT_4_R	
Forward	AGTTCCAACGAGGGTTTCAGG
Reverse	CAGCAGGTTGCCCAAGATG
5-HT_5A_R	
Forward	ATGGATCTGCCTGTAAACTTGAC
Reverse	CACTCGGAAAGCTGAGAGAAAA
5-HT_5B_R	
Forward	TTGCTGATCGCTGCCACTTT
Reverse	GTCGAGGCCACCAAGTTATGT
5-HT_6_R	
Forward	GCTGTGCGTGGTCATCGTA
Reverse	CATCAGGTCCGACGTGAAGAG
5-HT_7_R	
Forward	CCGACCTCTACGGCCATCT
Reverse	TCTCGACTCTGCCATAGTTGAT

### Statistical analyses

All quantitative results are expressed as means ± SEM. Data were plotted and analyzed statistically using the GraphPad Prism 8.3.0 software. Data were analyzed with Student’s *t*-test, or ANOVA (one-way and two-way) followed by post-hoc analyses for multiple comparisons. Offline analysis of the data of electrophysiological recordings was conducted using the Clampfit software version 10.7 (Axon Instruments, Inc., CA) and Mini Analysis software version 6.03. *P* < 0.05 was considered statistically significant.

## Results

### The projection of DRN^5-HT^ neurons to dCA1 was impaired in 5×FAD mouse brain

**Fig. 1:**
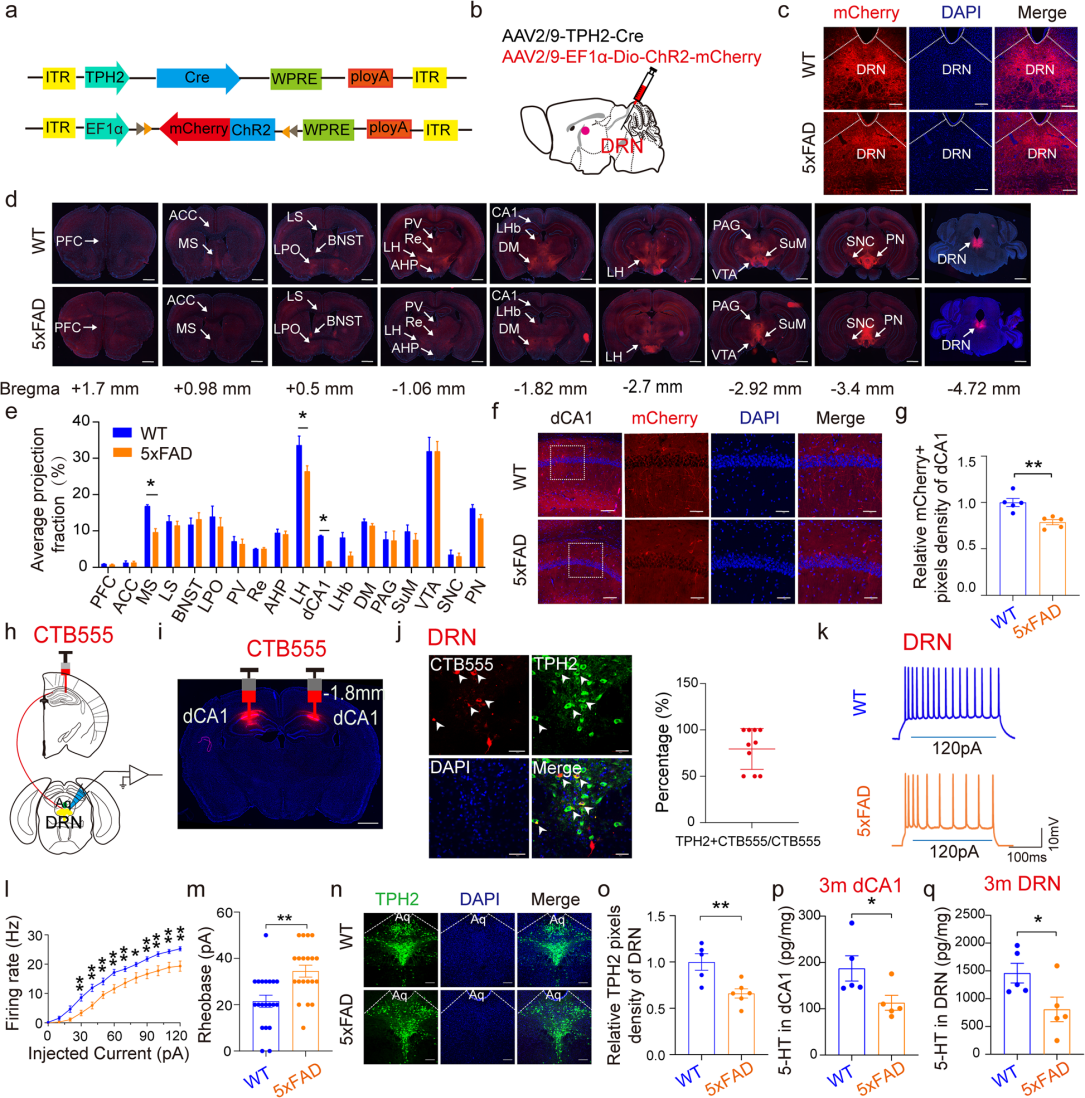
Whole-brain mapping of direct outputs from DRN^5-HT^ neurons. **a** Schematic diagram of AAV vector constructs. **b** Schematic of AAV-EF1α-Dio-hChR2(H134R)-mCherry combined with AAV-TPH2-Cre injection into the DRN. **c** Representation of mCherry expression in DRN. mCherry (red), DAPI (blue). Scale bar, 200 μm. **d** Representation of whole brain projection at nine coronal levels from DRN^5-HT^ neuron populations. Measurements are given in millimeters from the bregma. Scale bars, 1 mm. **e** Quantitative pixel density of output axons of DRN^5-HT^ neurons in the corresponding brain area. These data are expressed as the mean ± SEM, *n* = 3 per group, *P < 0.05, by two-tailed Student’s* t*-test. **f** Representation of DRN^5-HT^ neurons projecting into the dCA1. mCherry (red), DAPI (blue). Scale bars, 200 μm (left) and 50 μm (right). **g** Quantitative analysis of mCherry signals in the dCA1 region. WT = 5 mice, 5×FAD = 5 mice. These data are expressed as the mean ± SEM, **P < 0.01, by two-tailed Student’s* t*-test. **h** Schematic diagram of the CTB-555 virus injection site and electrophysiological recording of DRN^5-HT^-dCA1 neurons. **i** Representation of the virus injection site of dCA1. Scale bar, 1 mm. **j** Representative images of DRN^5-HT^-dCA1 neurons (yellow) labeled by CTB-555 and TPH2 in DRN. CTB-555 (red), TPH2 (green), DAPI (blue). Scale bars, 50 μm. Percentage of CTB-555-labeled neurons that expressed TPH2 in the DRN (*n* = 10 cells from 6 mice). **k** Representative traces from DRN^5-HT^-dCA1 neurons. **l** Quantification of firing rates induced by current injection recorded from DRN^5-HT^-dCA1 neurons. WT = 20 cells from 5 mice, 5×FAD = 20 cells from 5 mice. The data are expressed as the mean ± SEM, *P < 0.05, by two-tailed Student’s* t*-test. **m** Quantification of rheobase to evoke an action potential under current step recorded from DRN^5-HT^-dCA1 neurons. WT = 20 cells / 5 mice, 5×FAD = 20 cells / 5 mice. The data are expressed as the mean ± SEM, *P < 0.05, by two-tailed Student’s* t*-test. **n** Representation of TPH2 expression in DRN. TPH2 (green), DAPI (blue). Scale bars, 200 μm. **o** Quantitative analysis of TPH2 density in DRN. WT = 5 mice, 5×FAD = 6 mice. These data are displayed as mean ± SEM, **P < 0.01, ns, no significance, by two-tailed Student’s* t*-test. **p** Quantitative analysis of 5-HT in the hippocampus. The data are expressed as the mean ± SEM, *n* = 5 per group, *P < 0.05, by two-tailed Student’s* t*-test. **q** Quantitative analysis of 5-HT in the DRN. The data are expressed as the mean ± SEM, *n* = 5 per group, *P < 0.05, by two-tailed Student’s* t*-test

DRN^5-HT^ neurons have extensive projections to lots of brain regions which mediate diverse neuronal functions. Considering the vital role of DRN^5-HT^ neurons in depression and cognition, we explored the neuronal projection of DRN^5-HT^ neurons in early 5×FAD mouse brains. The mixture of AAV2/9-EF1α-Dio-mCherry and AAV2/9-TPH2-Cre viruses was injected into the DRN region of 2-month-old WT and 5×FAD mice to monitor the neuronal projection of DRN^5-HT^ neurons (Fig. 1a, b). The virus expression was first confirmed by mCherry signals in DRN after 4 weeks of virus expression (Fig. 1c). Then, the projections of DRN^5-HT^ neurons in the whole brain were detected based on the mCherry fluorescence signal (Fig. 1d). The projection of DRN^5-HT^ neurons can be found in many brain regions (Fig. 1d, e). Among these regions, the DRN^5-HT^-positive axons in the medial septum (MS), lateral hypothalamus (LH), and dCA1 regions were significantly decreased in 5×FAD mice compared with WT mice (Fig. 1d, e). As an important brain region affected in AD, hippocampal dCA1 draws our attention. Further detailed analysis of neuronal fibers in dCA1 clearly indicated that the projections of DRN^5-HT^ neurons to dCA1 were selectivity decreased in 3-month-old 5×FAD mouse brain (Fig. 1f, g).

### DRN^5-HT^-dCA1 neuronal activity was decreased in early 5×FAD mice

Considering the decreased projection of DRN^5-HT^ neurons to dCA1 in early 5×FAD mouse brains, we wondered whether the excitability of DRN^5-HT^-dCA1 neurons also changed. To visualize the DRN^5-HT^-dCA1 connected neurons, the retrograde tracer cholera subunit B (CTB-555) was injected into the dCA1 area (Fig. 1h, i). As predicted, the retrograde CTB-555 signals were observed in the DRN 1 week after injection. Moreover, most of the CTB-555-positive neurons were co-labeled with TPH2, a marker of 5-HT-activated neurons (Fig. 1j). These data confirmed the DRN^5-HT^-dCA1 projection. By electrophysiological recording, we found that the DRN^5-HT^-dCA1 labeled neurons displayed decreased firing rate and increased rheobase in 5×FAD mice compared with WT, indicating the impaired neuronal activity of DRN^5-HT^-dCA1 neurons in early 5×FAD mice (Fig. 1k–m). Accompanying with the decreased neuronal activity, the expression of TPH2 was also found decreased in DRN and dCA1 regions from the early (3-month-old)- and middle (6-month-old)-stage 5×FAD mice compared with the age-matched WT controls (Fig. 1n, o and Fig. S1a–h). We performed staining for the cleaved-caspase-3 in the DRN of WT and 5×FAD mice, and found no differences (Fig. S1i). This result suggested that the decreased expression of TPH2 in the DRN of 5×FAD mice was not caused by neuronal death. In addition, intracellular 5-HT levels were also measured. Accompanying with the decreased TPH2 expression, the serotonin levels were found decreased in both dCA1 and DRN in 5×FAD mice (Fig. 1p, q).

### The DRN^5-HT^ neurons project to dCA1^CaMKII^ neurons

**Fig. 2:**
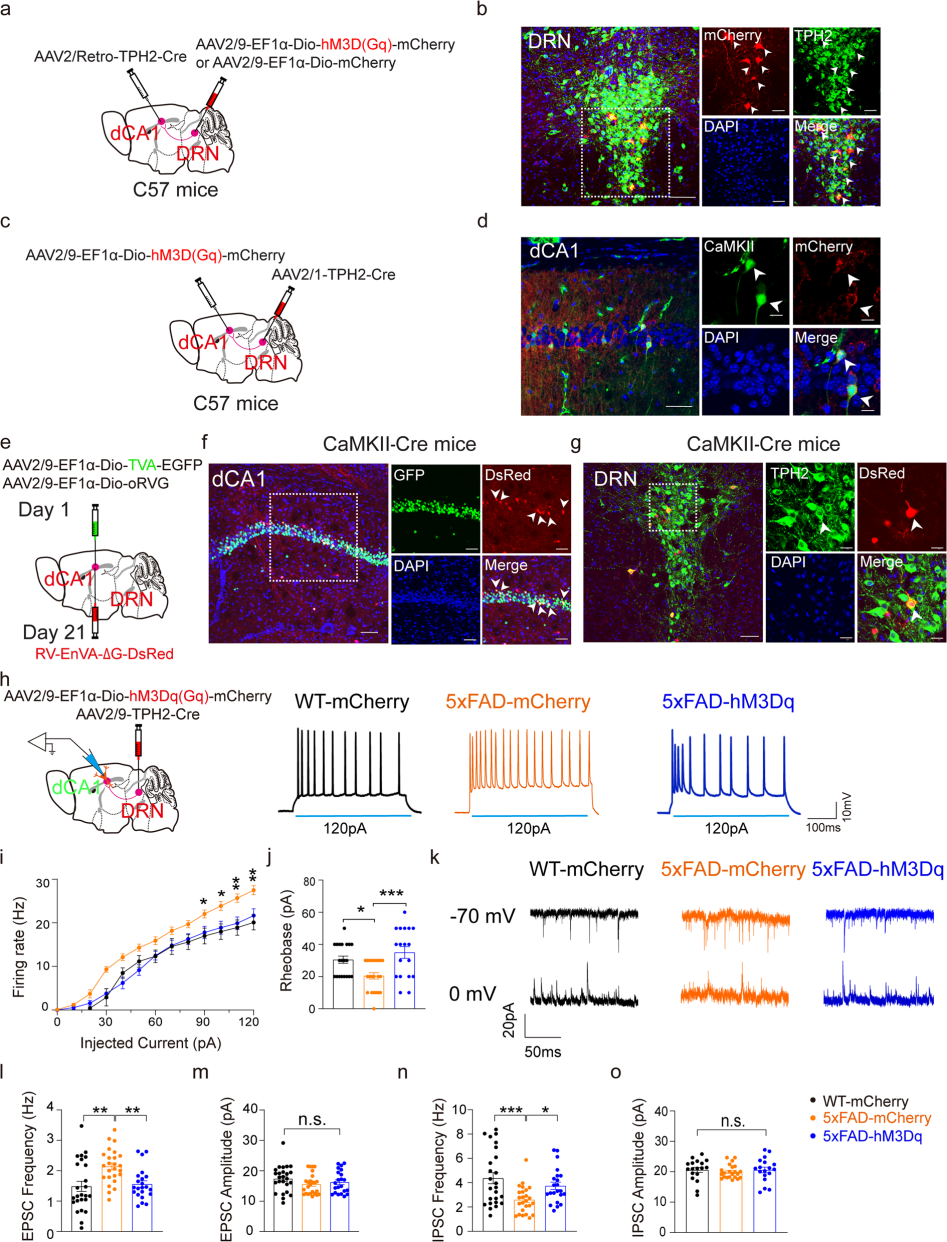
Structural analysis of the DRN^5-HT^-dCA1^CaMKII^ neural circuit. **a** Schematic of retrograde labeled virus tracing strategy. **b** The mCherry signals were co-localized with TPH2, a 5-HT neuronal marker, in the DRN. mCherry (red), TPH2 (green), DAPI (blue). Scale bars, 50 μm. **c** Schematic of anterograde labeled virus tracing strategy and electrophysiological recording of a DRN^5-HT^-dCA1^CaMKII^ neuron. **d** The mCherry signals were co-localized with CaMKII immunofluorescence in the dCA1. mCherry (red), CaMKII (green), DAPI (blue). Scale bar, 5 μm. **e** Schematic of the Cre-dependent retrograde trans-monosynaptic rabies virus tracing strategy. **f** Typical images of viral expression within the dCA1 of CaMKII-Cre mice. Starter cells (yellow) co-expressing AAV2/9-Dio-TVA-EGFP, AAV2/9-Dio-oRVG (green), and RV-EnVA-△G-dsRed (red). Scale bars, 100 μm (left) and 50 μm (right). **g** DsRed signals were co-localized with TPH2 immunofluorescence in the DRN of CaMKII-Cre mice. Scale bars, 100 μm (left) and 20 μm (right). **h** Representative traces showing the effects of chemogenetic activation of dCA1^5-HT^ terminals. **i**, **j** Quantification of firing rates of action potential and spike rheobase under current step recorded from DRN^5-HT^-dCA1^CaMKII^ neurons. WT-mCherry = 20 cells from 5 mice, 5×FAD-mCherry = 22 cells from 5 mice, 5×FAD-hM3Dq = 18 cells from 5 mice. The data are expressed as the mean ± SEM, *P < 0.05, by one-way ANOVA followed by the Holm-Sidak’s pairwise test. **k** Sample traces showing the effects of chemogenetic activation of dCA1^5-HT^ terminals on sEPSCs and sIPSCs recorded from CA1^CaMKII^ neurons at −70 mV and 0 mV. **l** Quantification of the frequency of sEPSCs recorded from dCA1^CaMKII^ neurons. WT-mCherry = 20 cells from 5 mice, 5×FAD-mCherry = 22 cells from 5 mice, 5×FAD-hM3Dq = 18 cells from 5 mice. The data are expressed as the mean ± SEM, **P < 0.01, n.s., by one-way ANOVA followed by the Holm-Sidak’s pairwise test. **m** Quantification of the amplitude of sEPSCs recorded from dCA1^CaMKII^ neurons. WT-mCherry = 20 cells from 5 mice, 5×FAD-mCherry = 22 cells from 5 mice, 5×FAD-hM3Dq = 18 cells from 5 mice. The data are expressed as the mean ± SEM, n.s., no significance, by one-way ANOVA followed by the Holm-Sidak’s pairwise test. **n** Quantification of the frequency of sIPSCs recorded from dCA1^CaMKII^ neurons. WT-mCherry = 20 cells from 5 mice, 5×FAD-mCherry = 22 cells from 5 mice, 5×FAD-hM3Dq = 18 cells from 5 mice. The data are expressed as the mean ± SEM, *P < 0.05, ***P < 0.001, by one-way ANOVA followed by the Holm-Sidak’s pairwise test. **o** Quantification of the amplitude of sIPSCs recorded from dCA1^CaMKII^ neurons. WT-mCherry = 20 cells from 5 mice, 5×FAD-mCherry = 22 cells from 5 mice, 5×FAD-hM3Dq = 18 cells from 5 mice. The data are expressed as the mean ± SEM, n.s., no significance, by one-way ANOVA followed by the Holm-Sidak’s pairwise test

To further confirm the direct DRN^5-HT^-dCA1 projection, two strategies were used. First, the AAV2/Retro-TPH2-Cre and AAV2/9-EF1α-Dio-mCherry virus was injected into the dCA1 and the DRN, respectively. We subsequently observed mCherry-expressing neurons in the DRN region which also can be labeled with TPH2. These data suggested direct synaptic connection of serotonin neurons in DRN with dCA1 (Fig. 2a, b). We then exchanged the viral infusion regions by injecting AAV2/1-TPH2-Cre virus into the DRN and AAV2/9-EF1α-Dio-mCherry into the dCA1 region of WT mice. We observed mCherry-expressing neurons in the dCA1, which were co-labeled with excitatory neuronal marker CaMKII (Fig. 2c, d and Fig. S2a, b) but not with inhibitory neuronal marker GAD67 (Fig. S2c).

To further characterize the DRN-dCA1 neuronal organization, CaMKII-Cre mice and GAD2-Cre mice were used for cell-type-specific retrograde trans-monosynaptic tracing. The Cre-dependent helper viruses (AAV2/9-EF1α-Dio-TVA-EGFP and AAV2/9-EF1α-Dio-oRVG) were injected into the dCA1 of CaMKII-Cre or GAD2-Cre mice. Three weeks later, the rabies virus (RV) (RV-EnVA-△G-dsRed) was injected into the same site (Fig. 2e and Fig. S2d). We observed that the DsRed signal was mostly co-localized with GFP in CaMKII-Cre mice (Fig. 2f) but not in GAD2-Cre mice (Fig. S2e). The presence of these helper viruses also enabled the RV to spread retrogradely across monosynapses. As predicted, we observed that the DsRed signal in the DRN was co-labeled with TPH2 immunostaining in CaMKII-Cre mice (Fig. 2g) but not in GAD2-Cre mice (Fig. S2f). These findings revealed a new neuronal circuit from DRN serotonin neurons to hippocampal dCA1 excitatory neurons.

### The hyperactivity of dCA1^CaMKII^ neurons was alleviated by the activation of the DRN^5-HT^-dCA1^CaMKII^ neural circuit in 5×FAD mice

To assess the role of the DRN^5- HT^-dCA1^CaMKII^ neural circuit in early 5×FAD mice, a chemogenetic strategy was used, injecting the AAV2/9-EF1α-Dio-hM3Dq (Gq)-mCherry virus into the DRN region, and the AAV2/9-TPH2-Cre virus into the dCA1 region. Whole-cell recordings of neurons in dCA1 were performed during chemogenetic activation of hM3Dq (Gq)-containing DRN^5-HT^ terminals in the dCA1. Enhanced firing rate and decreased rheobase were found in dCA1^CaMKII^ neurons in early 5×FAD mice (Fig. 2h–j), which were significantly alleviated by activating 5-HT axon terminals (Fig. 2h–j). The balance of excitation and inhibition synaptic transmission (E/I) is also related to psychiatric disorders [31]. Then, the sEPSCs/sIPSCs in dCA1^CaMKII^ neurons were recorded before and after activating 5-HT neurons. The frequency of sEPSCs and sIPSCs increased and decreased respectively in 3-month-old 5×FAD mice compared with WT. No obvious changes in the amplitudes of sEPSCs and sIPSCs were found. Chemogenetic activation of DRN^5-HT^ neurons projecting to dCA1^CaMKII^ neurons significantly reversed the changes of frequency of sEPSCs and sIPSCs in dCA1^CaMKII^ neurons of 5×FAD mice (Fig. 2k–o). These results suggest that the axons of 5-HT neurons in the DRN form synaptic connections with hippocampal dCA1 excitatory neurons, and the stimulation of DRN serotonergic neurons produced a rapid inhibition of hippocampal excitatory neurons and reversed the impairment of synaptic transmission. Furthermore, the LTP impairments in the early 5×FAD mice were also reversed by chemogenetic activation of the DRN^5-HT^-dCA1^CaMKII^ neural circuit (Additional file 1: Fig. S3a, b), suggesting that this neural circuit not only affects neuronal activity but also affects synaptic transmission in hippocampal dCA1.

### Chemogenetic activation of the DRN^5-HT^-dCA1^CaMKII^ neural circuit rescued depressive symptoms and subsequent cognitive impairments in 5×FAD mice

**Fig. 3:**
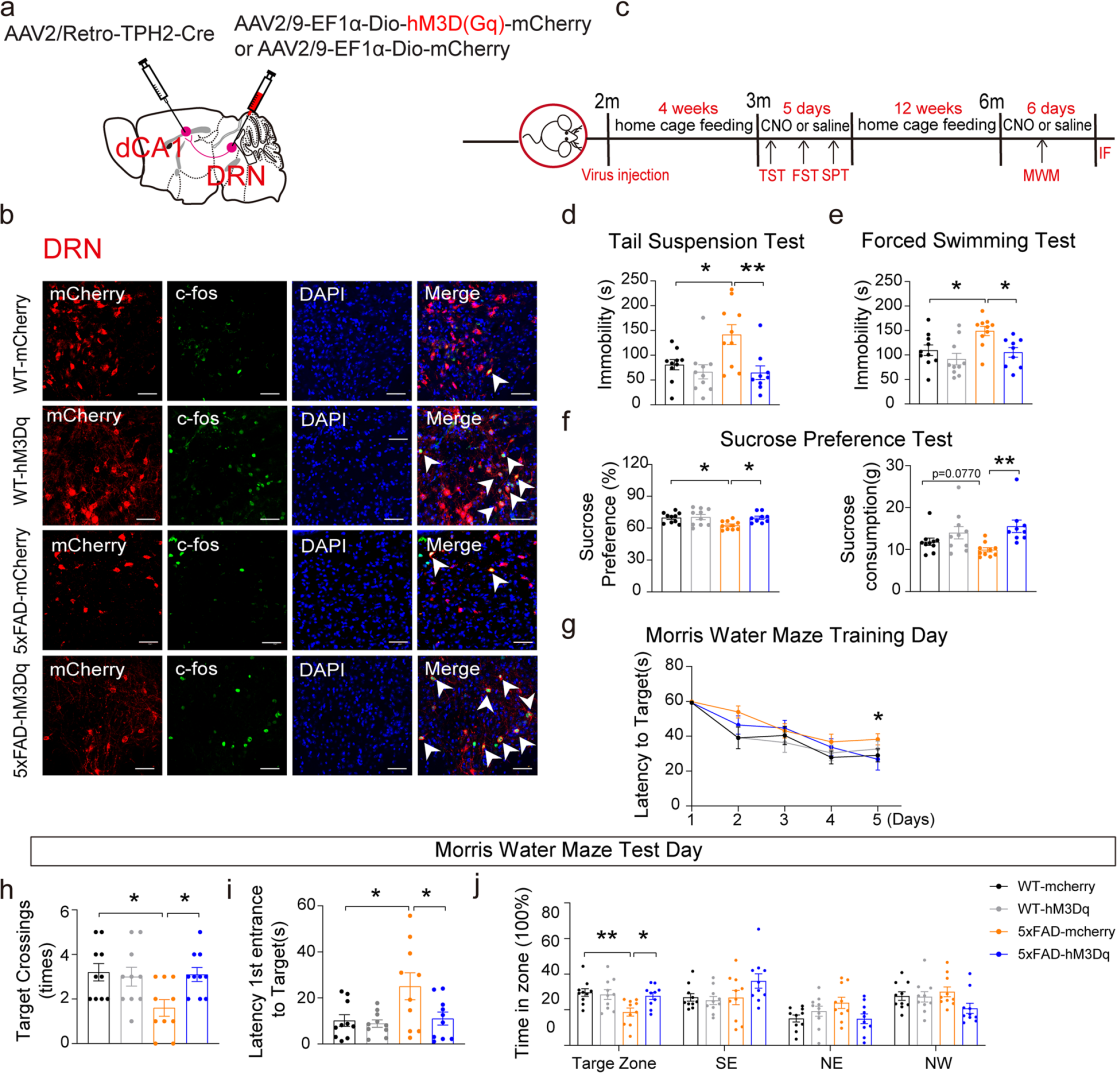
Chemogenetic activation of DRN^5-HT^ neurons in the DRN-dCA1 neural circuit attenuated depressive symptoms and cognitive impairments in 5×FAD mice. **a** Schematic of retrograde labeled virus tracing strategy. **b** The mCherry signals were co-localized with c-fos immunofluorescence in the DRN. mCherry (red), c-fos (green), DAPI (blue). Scale bars, 50 μm. **c** Schematic of chemogenetic manipulations. **d**-**f** Quantification of chemogenetic activation of DRN^5-HT^-dCA1^CaMKII^ neural circuit by TST, FST, and SPT. WT-mCherry (*n* = 10), WT-hM3Dq (*n* = 10), 5×FAD-mCherry (*n* = 10), 5×FAD-hM3Dq (*n* = 10). The data are expressed as the mean ± SEM, *P < 0.05, **P < 0.01, by one-way ANOVA followed by Holm-Sidak’s pairwise test. **g** Latency to target is recorded during the 5-day training stage. **h**-**j** MWM analysis as target crossings, latency to the target (s), and time in zone (%) in the invisible platform tests. WT-mCherry (*n* = 10), WT-hM3Dq (*n* = 10), 5×FAD-mCherry (*n* = 10), 5×FAD-hM3Dq (*n* = 10). The data are expressed as the mean ± SEM, *P < 0.05, **P < 0.01, by one-way ANOVA followed by the Holm-Sidak’s pairwise test

The above data indicated that activation of the DRN^5-HT^-dCA1^CaMKII^ neural circuit would increase the hippocampal synaptic transmission in early 5×FAD mice. Then, the effects of this circuit on the depressive behaviors were tested. The AAV2/Retro-TPH2-Cre virus was injected into the dCA1 region, and AAV2/9-EF1α-Dio-hM3Dq (Gq)-mCherry or AAV2/9-EF1α-Dio-mCherry (serving as the control AAV) was injected into the DRN region of WT and 5×FAD mice (Fig. 3a). Enhanced mCherry/c-Fos double labeling of neurons after CNO administration firstly proved the success of this strategy (Fig. 3b).

The 3-month-old 5×FAD and WT mice that received the AAV were subjected to behavioral tests (Fig. 3c). Before the cognitive impairments occur, the 3-month-old 5×FAD mice exhibited significant depressive behaviors as evaluated by TST, FST, and SPT (Fig. 3d–f), but not anxiety-like behaviors (Fig. S4a–c). Activation of the DRN^5-HT^-dCA1^CaMKII^ neural circuit significantly rescued the depressive-like behaviors in early 5×FAD mice (Fig. 3d–f). Depressive symptoms are also a risk factor for AD and precede cognitive impairments. These mice further underwent the MWM test at 6 months of age. Activation of the DRN^5-HT^-dCA1^CaMKII^ neural circuit significantly attenuated the cognitive impairments in 5×FAD mice during the training stage (Fig. 3g) and on the test day (Fig. 3h–j). Specifically, 5×FAD-hM3Dq mice displayed more crossings of the platform, reduced latency to reach the platform, and more residence time in the target quadrant, compared with 5×FAD-mCherry mice (Fig. 3h–j). The mCherry^+^  neurons in the DRN were sensitive to 10-μM CNO stimuli (Fig. S5a, c), and they were 5-HT neurons (Fig. 2b). Together, these data suggested that the DRN^5-HT^-dCA1^CaMKII^ neural circuit plays antidepressant roles in early 5×FAD mice and attenuates cognitive impairment of 5×FAD mice at later stages.

### Optogenetic activation of the DRN^5-HT^-dCA1^CaMKII^ neural circuit rescued depressive symptoms and cognitive impairments in 5×FAD mice

**Fig. 4:**
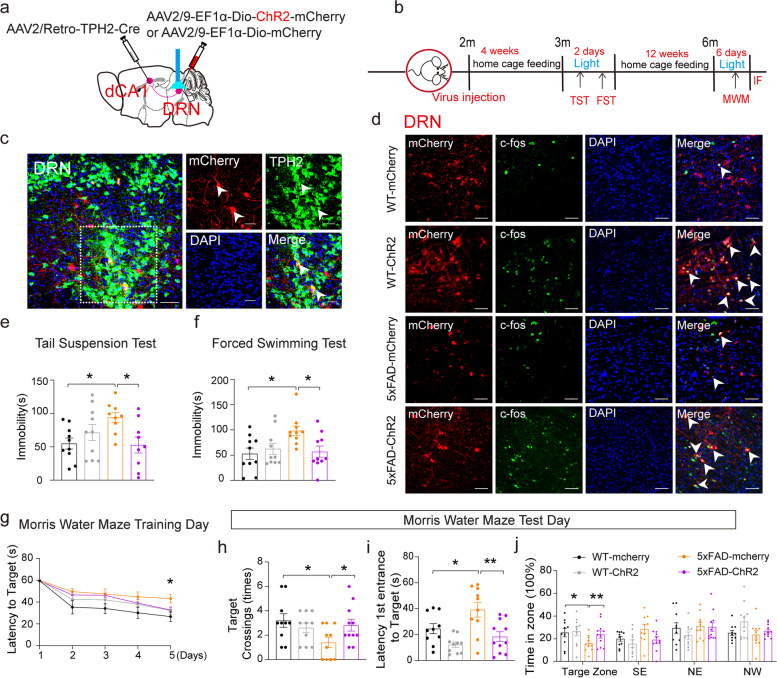
Optogenetic activation of DRN^5-HT^ neurons in the DRN-dCA1 neural circuit improved depressive symptoms and cognitive impairment of 5×FAD mice. **a** Schematic of retrograde labeled virus tracing strategy and optogenetic manipulation. **b** Schematic of optogenetic manipulation. **c** The mCherry signals were co-localized with 5-HT neuronal marker TPH2 immunofluorescence in the DRN. mCherry (red), TPH2 (green), DAPI (blue). Scale bars, 50 μm. **d** The mCherry signals were co-localized with c-fos immunofluorescence in the DRN. mCherry (red), c-fos (green), DAPI (blue). Scale bars, 50 μm. **e**, **f** Effect of optogenetic activation of DRN^5-HT^-dCA1^CaMKII^ neural circuit  on performance in TST and FST. WT-mCherry (*n* = 10), WT-ChR2 (*n* = 10), 5×FAD-mCherry (*n* = 10), 5×FAD-ChR2 (*n* = 11). The data are expressed as the mean ± SEM, *P < 0.05, by one-way ANOVA followed by Holm-Sidak’s pairwise test. **g** Latency to target recorded during the 5-day training stage. **h**-**j** Number of crossings, the latency to arrive at the platform (s), and the time in the zone during the MWM test phase. WT-mCherry (*n* = 10), WT-ChR2 (*n* = 10), 5×FAD-mCherry (*n* = 10), 5×FAD-ChR2 (*n* = 11). The data are expressed as the mean ± SEM, *P < 0.05, **P < 0.01, by one-way ANOVA followed by Holm-Sidak’s pairwise test

To strengthen our finding from chemogenetic modulations in 5×FAD mice, optogenetic manipulations were also performed. The AAV2/Retro-TPH2-Cre virus was injected into the dCA1 region and the AAV2/9-EF1α-Dio-ChR2-mCherry virus was injected into the DRN of 2-month-old WT and 5×FAD mice, with the optical fibers implanted above the DRN region (Fig. 4a). The depressive and cognitive behaviors of these mice were measured at 3 and 6 months of age, respectively (Fig. 4b). We first confirmed the activation of DRN^5-HT^ neurons (Fig. 4c) indicated by the mCherry/c-Fos double labeling after optogenetic activation (Fig. 4d). Consistent with the chemogenetic manipulation data, optogenetic activation of the DRN^5-HT^-dCA1 neurons also significantly alleviated the depressive-like behavior in early 3-month-old 5×FAD mice (Fig. 4e, f), and attenuated the cognitive impairments in 6-month-old 5×FAD mice (Fig. 4g–j). The mCherry^+^  neurons in the DRN were sensitive to 473 nm light stimuli (Fig. S5b, d), and they were 5-HT neurons (Fig. 4c).

### Agonists of 5-HT_1B_R and 5-HT_4_R reduced the activity of dCA1^CaMKII^ neurons and improved depressive symptoms and cognitive impairments in 5×FAD mice

**Fig. 5:**
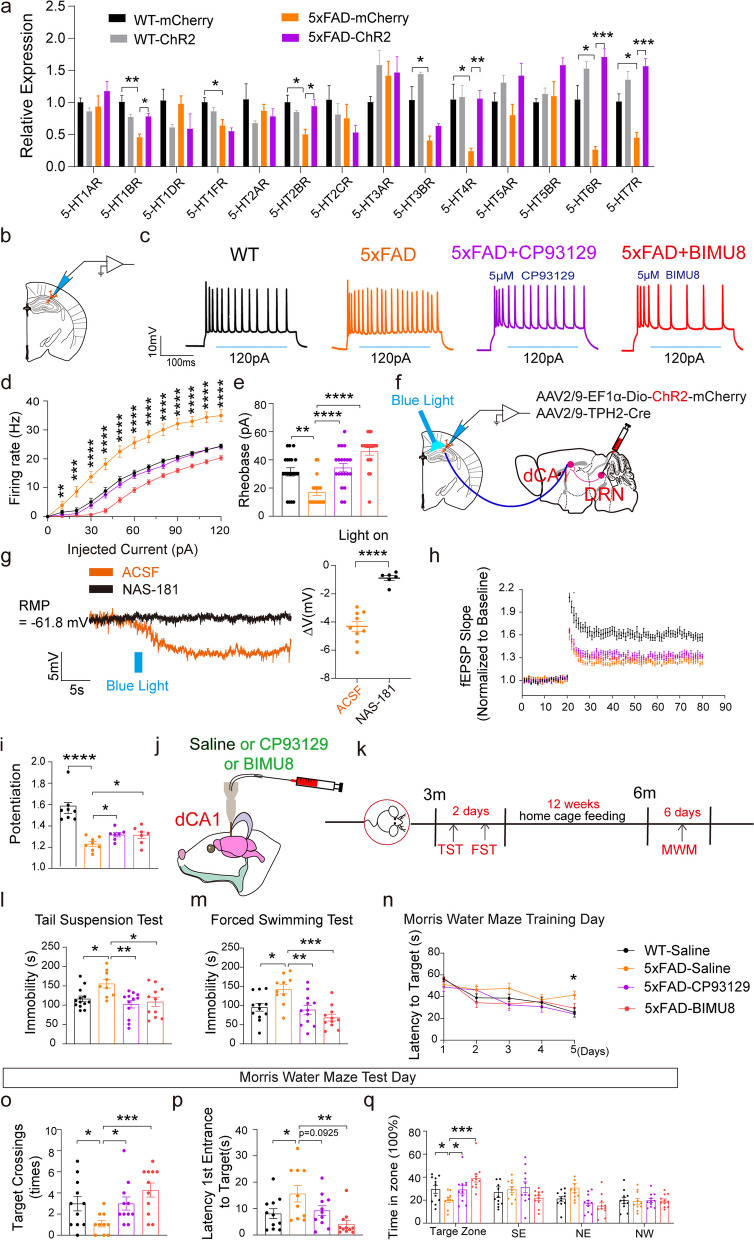
5-HT_1B_R agonist or 5-HT_4_R agonist alone reduced the activity of dCA1^CaMKII^ neurons, and improved depressive symptoms and cognitive impairment in 5×FAD mice. **a** Effect of optogenetic activation of DRN^5-HT^ neurons in the DRN-dCA1 neural circuit on the relative expression of 5-HT receptors from mRNA analysis. The data are expressed as the mean ± SEM, *P < 0.05, **P < 0.01, ***P < 0.001, by one-way ANOVA followed by the Holm-Sidak’s pairwise test. **b** Recording configuration in acute slices. **c** Representative traces from dCA1^CaMKII^ neurons. **d**, **e** Quantification of firing rates of action potentials and spike rheobase under current step recorded from dCA1^CaMKII^ neurons. WT = 20 cells from 5 mice, 5×FAD = 20 cells from 5 mice, 5×FAD-CP93129 = 20 cells from 5 mice, 5×FAD-BIMU8 = 20 cells from 5 mice. The data are expressed as the mean ± SEM, **P < 0.05, ***P < 0.001, ****P < 0.0001, by one-way ANOVA followed by the Holm-Sidak’s pairwise test. **f** Schematic showing the injection of AAV2/9-Dio-ChR2-mCherry and AAV2/9-TPH2-Cre into the DRN and whole-cell patch recording of dCA1 neurons. **g** Representative traces and summarized data showing hyperpolarized potentials (*V*) in dCA1^CaMKII^ neurons evoked by photostimulation of DRN^5-HT^ terminals in the dCA1 in the presence of ACSF or 5-HT_1B_ receptor antagonist NAS-181. ACSF = 9 cells from 4 mice, NAS-181 = 6 cells from 3 mice. The data are expressed as the mean ± SEM, ****P < 0.0001, by two-tailed Student’s* t*-test. **h** LTP was induced by two trains of 100-Hz stimuli in the Schaffer collaterals. **i** Significant differences in fEPSP potentiation were determined by comparing fEPSP slopes during the last 10 min of recording after high-frequency stimulation. WT = 7 slices from 5 mice, 5×FAD = 8 slices from 5 mice, 5×FAD-CP93129 = 7 slices from 5 mice, 5×FAD-BIMU8 = 7 slices from 5 mice. The data are expressed as the mean ± SEM, *P < 0.05, ****P < 0.0001, by one-way ANOVA followed by the Holm-Sidak’s pairwise test. **j** Schematic paradigm of drug administration. **k** Timeline of experiments. **l**, **m** Effect of 5-HT_1B_R agonist CP93129 and 5-HT_4_R agonist BIMU8 on freezing time in TST and FST. WT-Saline (*n* = 13), 5×FAD-Saline (*n* = 9), 5×FAD-CP93129 (*n* = 12), 5×FAD-BIMU8 (*n* = 11). The data are expressed as the mean ± SEM, *P < 0.05, **P < 0.01, ***P < 0.001, by one-way ANOVA followed by Holm-Sidak’s pairwise test. **n** Effects of 5-HTR_1B_ agonist CP93129 and 5-HTR_4_ agonist BIMU8 on the latency to the target in the water maze training phase. **o**-**q** Effect of 5-HT_1B_R agonist CP93129 and 5-HT_4_R agonist BIMU8 on the number of crossings of the platform, the latency to the platform (s), and the time in the zone during the MWM test phase. WT-Saline (*n* = 13), 5×FAD-Saline (*n* = 9), 5×FAD-CP93129 (*n* = 12), 5×FAD-BIMU8 (*n* = 11). The data are expressed as the mean ± SEM, *P < 0.05, **P < 0.01, ***P < 0.001, by one-way ANOVA followed by Holm-Sidak’s pairwise test

The implications of the DRN^5-HT^-dCA1^CaMKII^ neural circuit in 5×FAD prompted us to explore whether the 5-HT receptors (5-HTRs) are also involved in this regulation. We examined the mRNA levels of 14 subtypes of 5-HTRs in dCA1 from the chemogenetically or optogenetically manipulated WT and 5×FAD mice mentioned above. Notably, the mRNA levels of 5-HT_1B_R, 5-HT_1F_R, 5-HT_2B_R, 5-HT_3B_R, 5-HT_4_R, 5-HT_6_R and 5-HT_7_R were significantly decreased in the dCA1 of 5 FAD mice compared with WT. The down-regulation of these 5-HTR genes was reversed after optogenetic or chemogenetic activation of the DRN^5-HT^-dCA1^CaMKII^ neural circuit (Fig. 5a, Fig. S6a) manipulation. Among the seven changed 5-HTRs, the 5-HT_1B_R and 5-HT_4_R were selected in our study for the following reasons: 5-HT_1B_R and 5-HT_4_R are highly expressed in the HPC and participate in the regulation of the hippocampal neuronal activity [24, 32]. 5-HT_1B_R [16, 33] and 5-HT_4_R [34–36] have been considered as drug targets for serotonergic system-related diseases, and their activation has antidepressant and neuroprotective effects [37, 38].

Next, 5-HT_1B_R agonist CP93129 and 5-HT_4_R agonist BIMU8 were used to activate the corresponding receptors. Consistent with the chemogenetic data in Fig. 2, the hyperexcitability of hippocampal dCA1^CaMKII^ neurons in 5×FAD mice was significantly reversed by CP93129 or BIMU8 (Fig. 5b–e). The brief blue light stimulation of ChR2-containing DRN^5-HT^ terminals in the dCA1 elicited a hyperpolarized potential in dCA1^CaMKII^ neurons, which was reversed by the application of the 5-HT_1B_R antagonist NAS-181(Fig. 5f, g). Of note, the hyperpolarized potential was not affected in the presence of the GABA_A_ receptor antagonist picrotoxin (PTX, Tocris Bioscience, Cat# 1128) (Fig. S7a–d), suggesting that the 5-HT_1B_R mediated fast, transient, GABA-independent hyperpolarization of dCA1 excitatory neurons. Furthermore, the activation of 5-HT_1B_R or 5-HT_4_R also attenuated the hippocampal LTP deficits in 5×FAD mice (Fig. 5h, i). More importantly, direct administration of 5-HT_1B_R agonist CP93129 or 5-HT_4_R agonist BIMU8 into the dCA1 via cannular infusion (Fig. 5j, k) significantly relieved the depressive behaviors in 3-month-old 5×FAD mice (Fig. 5l, m), and improved cognitive impairments in 6-month-old 5×FAD mice (Fig. 5n–q). In addition, Nissl staining revealed no differences among these four groups, suggesting that the injection dose has little effects on the anatomical changes (Fig. S7e).

### Up-regulation of TPH2 in DRN^5-HT^ neurons attenuated the depressive symptoms and cognitive impairments in 5×FAD mice

**Fig. 6:**
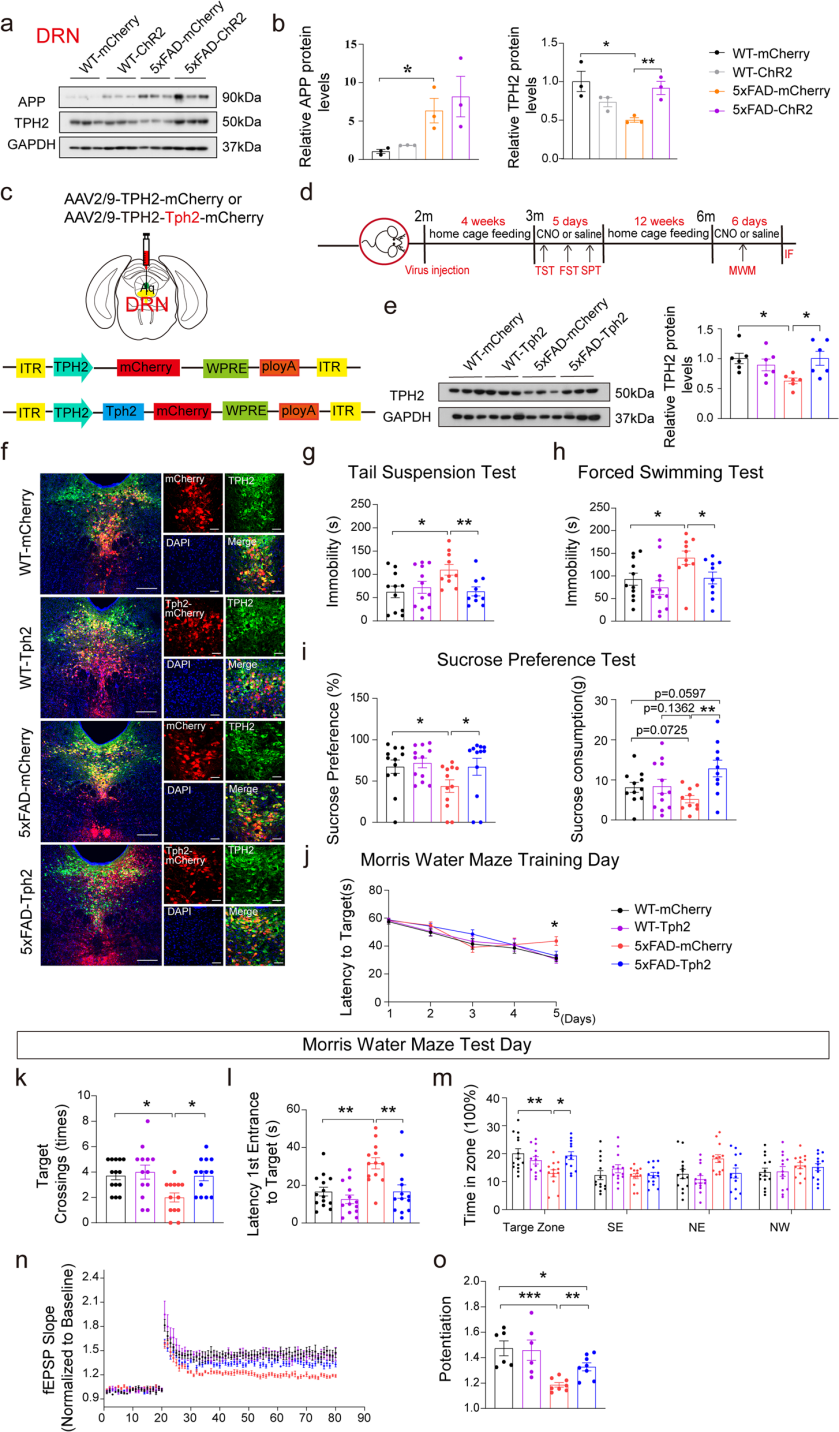
TPH2 overexpression rescued early depressive symptoms and later cognitive impairment in 5×FAD mice. **a** Representation of TPH2 expression in the DRN. **b** Quantitative analysis of APP and TPH2 expression in DRN. The data are expressed as the mean ± SEM, *n* = 3 per group, *P < 0.05, **P < 0.01, by one-way ANOVA followed by Holm-Sidak’s pairwise test. **c** Schematic representation of DRN infusion sites and virus strategy. **d** Timeline of experimental design. **e** The TPH2 protein levels in mouse brains injected with AAV-Tph2-mCherry or control AAV-mCherry were determined by western blotting. The data are expressed as the mean ± SEM, *n* = 6 per group, *P < 0.05, by one-way ANOVA followed by Holm-Sidak’s pairwise test. **f** The mCherry signals were co-localized with 5-HT neuronal marker TPH2 in the DRN. mCherry (red), TPH2 (green), DAPI (blue). Scale bar, 50 μm. **g**-**i** Effect of TPH2 overexpression on depressive symptoms in TST, FST, and SPT. WT-mCherry (*n* = 11), WT- Tph2 (*n* = 12), 5×FAD-mCherry (*n* = 10), 5×FAD- Tph2 (*n* = 10). The data are expressed as the mean ± SEM, *P < 0.05, **P < 0.01, by one-way ANOVA followed by Holm-Sidak’s pairwise test. **j** Effects of TPH2 overexpression on the latency to the target in the water maze training phase. **k**-**m** Effect of TPH2 overexpression on the number of crossings of the platform, latency to the platform (s), and the time in the zone during the MWM test phase. WT-mCherry (*n* = 11), WT-Tph2 (*n* = 12), 5×FAD-mCherry (*n* = 10), 5×FAD-Tph2 (*n* = 10). The data are expressed as the mean ± SEM, *P < 0.05, **P < 0.01, by one-way ANOVA followed by Holm-Sidak’s pairwise test. **n** LTP was induced by two trains of 100-Hz stimuli in the Schaffer collaterals. **o** Significant differences in fEPSP potentiation were determined by comparing fEPSP slopes during the last 10 min of recording after high-frequency stimulation. WT-mCherry = 6 slices from 6 mice, WT-Tph2 = 6 slices from 6 mice, 5×FAD-mCherry = 8 slices from 6 mice, 5×FAD-Tph2 = 8 slices from 6 mice. These data are expressed as the mean ± SEM, *P < 0.05, ****P < 0.0001, by one-way ANOVA followed by Holm-Sidak’s pairwise test

The down-regulation of TPH2 expression (Fig. 1n–o, Fig. S1a–h) decreases 5-HT levels in 5×FAD mice and promotes depression [39–42]. We found that optogenetic activation of the DRN^5-HT^-dCA1^CaMKII^ neural circuit restored the expression of TPH2 in the DRN of 5×FAD mice (Fig. 6a, b). We speculated that the TPH2 level is crucial during these regulations. A *Tph2* promotor-derived TPH2 virus (AAV-Tph2-mCherry) and control AAV (AAV-mCherry) were constructed and injected into the DRN of 2-month-old WT and 5×FAD mice (Fig. 6c). These mice were subjected to tests as indicated in Fig. 6d. Enhanced expression of TPH2 and double labeling of THP2 with mCherry confirmed the TPH2 expression efficiency and specificity (Fig. 6e, f). Behavioral tests indicated that the overexpression of TPH2 in DRN significantly attenuated the depressive-like behaviors of 3-month-old 5×FAD mice measured by TST, FST, and SPT (Fig. 6g–i). After 3 months, the TPH2-overexpressing 5×FAD mice also showed improved learning and memory ability compared with 5×FAD mice injected with control AAV (Fig. 6j–m). Electrophysiological recording further indicated that the overexpression of TPH2 reversed the hippocampal-dependent LTP damage (Fig. 6n, o). Meanwhile, we did not observe significant differences in behavior tests between WT mice injected with TPH2-AAV and control AAV (Fig. 6g–m). Taken together, these data suggested that maintaining normal TPH2 levels in DRN is antidepressant and improves cognitive impairment in 5×FAD mice.

## Discussion

**Fig. 7:**
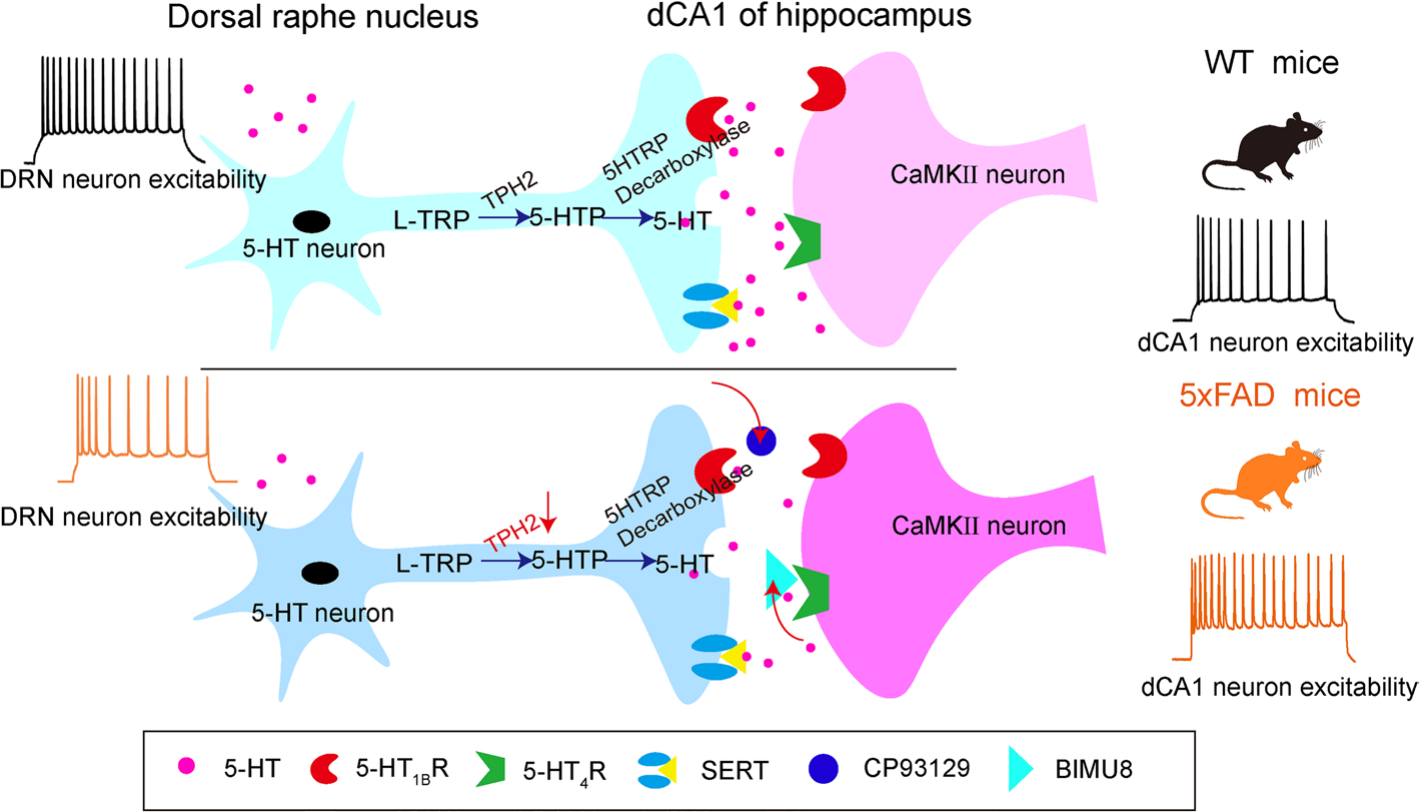
Schematic diagram showing that the DRN^5-HT^-dCA1^CaMKII^ neural circuit contributes to depressive symptoms and cognitive impairments in 5×FAD mice. In the AD state, decreased presynaptic 5-HT release and decreased 5-HT neuronal activity lead to hyperexcitability of postsynaptic CaMKII neurons by attenuating the functions of 5-HT_1B_R and 5-HT_4_R, resulting in increased glutamate toxicity and LTP impairment in the DRN^5-HT^-dCA1^CaMKII^ neural circuit. Activation of the DRN^5-HT^-dCA1^CaMKII^ neural circuit alleviates hyperexcitation of CaMKII neurons by enhancing the functions of 5-HT_1B_R and 5-HT_4_R, thereby reversing LTP damage and improving depressive symptoms and cognitive impairments in 5×FAD mice

5-HT neurons from the DRN widely project to diverse brain regions and are involved in the regulation of emotion and cognition in AD [24, 43–47]. In this study, we reveal a novel neural circuit underlying the early depressive symptoms and later cognitive impairments in 5×FAD model mice. We found that the excitability of DRN^5-HT^ neurons decreased and their projection to dCA1 was reduced in the 3-month-old 5×FAD mice. Activation of the DRN^5-HT^-CA1^CaMKII^ circuit by optogenetic or chemogenetic manipulations can alleviate the depressive behaviors in early-stage 5×FAD mice and the cognitive impairments in later-stage 5×FAD mice (Fig. 7). The DRN^5-HT^ mediates the inhibition of CA1^CaMKII^ neurons predominantly through the postsynaptic hyperpolarization of 5-HT_1B_R and depolarization of 5-HT_4_R. These results suggest that dCA1^CaMKII^ neurons are regulated by both 5-HT_1B_R and 5-HT_4_R, resulting in glutamate toxicity in 5×FAD model mice.

Soluble Aβ could induce glutamatergic hyperexcitability of hippocampal pyramidal neurons in early AD mice [48, 49]. Previous studies suggested that serotonergic signaling is reduced in the HPC of AD patients and mice [11, 49]. Here, we also found that the excitability of pyramidal neurons increased in early 5×FAD mice. However, the decreased excitability of DRN^5-HT^ neurons results in impaired nerve fiber projection to the HPC. The increased serotonergic input may act as a compensatory mechanism for maintaining synaptic activity, and alter the hippocampal glutaminergic circuits [50]. The hyperexcitability of dCA1 pyramidal neurons is closely related with the impairments of the serotonergic system, although the detailed mechanism needs further investigation. In addition, the decreased expression of 5-HT_1B_R and 5-HT_4_R in the HPC of AD patients and AD mice may also be related to the hyperexcitability of CA1 pyramidal neurons [51, 52]. Both 5-HT_1B_R agonists and 5-HT_4_R agonists may shift the cleavage of APP to the production of sAPPα, thus inhibiting amyloid formation, further reducing the hyperexcitability of CA1 pyramidal neurons and improving cognitive function in AD mice [51, 53].

Depression is one of the most pronounced clinical symptoms and one of the risk factors for AD [54]. Our animal studies also confirmed the emergence of depressive symptoms in 3-month-old 5×FAD mice, followed by cognitive decline at approximately 6 months old. We predicted that the decrease of 5-HT underlies the role of serotonergic system in depression-like phenotype and cognitive impairment [55, 56]. TPH2 is the main regulator for 5-HT generation in the brain. We found that the 5-HT level and TPH2 expression were both reduced in DRN and dCA1 in early 5×FAD mice, which is consistent with findings in AD postmortem brain tissues [57]. 5-HT neurons are mainly located in the DRN and MRN, which have a close projective connection with the HPC [58]. Our study confirmed that the DRN^5-HT^ neurons have a direct projection to dCA1. The specific activation of the DRN^5-HT^-dCA1^CaMKII^ neural circuit attenuated depressive symptoms and cognitive impairments in 5×FAD mice. A previous report demonstrated that 5-HT neurons from MRN can project to the dorsal hippocampus (dHPC), and activation of MRN^5-HT^-dHPC^CaMKII^ induces anxiety-like behaviors in mice [14]. The difference in behavioral results induced by the activation of the DRN or the MRN is related to the differential projections from the DRN and MRN. The ventral HPC is known to be implicated in anxiety [59] and receive more serotonergic projections from the MRN than the DRN [60]. The ventral tegmental area/substantia nigra (VTA/SN), the amygdala, the dHPC, and the septum are reported to receive more serotonergic projections from the DRN [60] and are involved in antidepressant-like effects [61]. Combined with our findings, the 5-HT neurons from the DRN and the MRN may play different roles in the regulation of emotion, which deserves further investigation.

5-HTRs are the core of the serotonergic system, regulating the functions of 5-HT neuron-projected brain regions [43, 47]. There are about 14 subtypes of 5-HTRs, most of which are GPCRs. Regulation of the serotonin system is complicated, which includes but is not limited to changes of the density of 5-HT neurons, their projection in the raphe nuclei, 5-HT metabolism, and expression of 5-HT uptake transporter [62]. In physiological conditions, the 5-HT system is at an equilibrium state. Our data indicated that the photogenetic activation of the DRN^5-HT^-dCA1^CaMKII^ neural circuit did not affect the mRNA expression 5-HTRs in WT mice. It is speculated that the density and the projection of 5-HT neurons in the DRN of WT mice are not affected. The decreases of 5-HT_1B_R, 5-HT_2B_R, 5-HT_3B_R, 5-HT_4_R, 5-HT_6_R, and 5-HT_7_R in the dCA1 of 5×FAD mice were significantly reversed after activation of the DRN^5-HT^-dCA1^CaMKII^ neural circuit. 5-HT_1B_R has been reported to have antidepressant effects in a depressive mouse model [63]. 5-HT_2B_R is expressed in both neurons and astrocytes to regulate the impulsivity/aggression behavior and antidepressant-like behavior, respectively [64]. 5-HT_4_R and 5-HT_6_R have been reported to have neuroprotective, neurotrophic, and cognitive-enhancing effects that could be beneficial in the treatment of AD [65]. 5-HT_7_R exerts antidepressant effects by forming the 5-HT_1A_R/5-HT_7_R heterodimeric complex [66]. Unlike other 5-HTRs, the 5-HT_3B_R is a ligand-gated ion channel. Activation of 5-HT_3B_R inhibits pyramidal neurons in the prefrontal cortex via GABAergic interneurons [67]. Here, we found that the hyperpolarized potential of the DRN^5-HT^-dCA1^CaMKII^ circuit was reversed by the 5-HT_1B_R antagonist NAS-181, but not by the GABA_A_ receptor blocker PTX. Although these upregulated 5-HTRs in our study have been found to display antidepressant-like effects through different ways in depressive model mice, whether they mediate AD-associated depression is unknown. Our data indicated that pharmacologically activating 5-HT_1B_R or 5-HT_4_R in dCA1 attenuated the early depressive symptoms and cognitive impairments in 5×FAD mice. However, neuronal excitation and inhibition by 5-HT through its receptors are very complicated. 5-HT_1A_R and 5-HT_3A_R are reported to be involved in the regulation of the excitability of CA1 pyramidal neurons in hAPP-J20 mice [49]. Our data demonstrated that 5-HT_1B_R and 5-HT_4_R are involved in the pathogenesis of AD. The underlying mechanisms of different 5-HT receptors deserve further investigations.

Presynaptic 5-HT regulates the release of neurotransmitters, and reduced glutamate release directly affects the functions of 5-HT_1A_R [58, 68, 69], 5-HT_1B_R [23, 70–72], and 5-HT_6_R [73]. Similarly, presynaptic inhibition of GABA release is also mediated by 5-HT_1A_R [74–76] and 5-HT_1B_R [77, 78]. Therefore, our results do not rule out the possibility of other 5-HTRs regulating mood and cognitive function. At least our data suggested that 5-HT_1B_R and 5-HT_4_R are more closely related to depressive symptoms in 5×FAD. Targeting 5-HT_1B_R and 5-HT_4_R will benefit AD patients with symptom relief.

Another common symptom in AD and major depression is neuroinflammation. During neuroinflammation, both the activated kynurenine pathway and the weakened 5-HT pathway are associated with tryptophan metabolism and deteriorate depression and cognitive impairment [79]. Kynurenine is metabolized to kynurenic acid, a primary neuroprotective metabolite, and quinolinic acid, a key neurotoxic metabolite [80]. TPH2, the key and rate-limited enzyme of serotonin synthesis in the brain, affects the risk of depression [81]. We found that the expression of TPH2 and the 5-HT levels were both reduced in DRN and dCA1 in early 5×FAD mice, which suggests that the shift of tryptophan metabolism to the kynurenine pathway leads to increased glutamate toxicity in the HPC. Activation of the DRN^5-HT^-CA1^CaMKII^ neural circuit is capable of increasing the expression of TPH2 in the DRN of 5×FAD mice. Consistent with this, TPH2 overexpression in DRN^5-HT^ neurons alleviated the damage of synaptic function in 5×FAD mice and attenuated the early depression and cognitive impairments. These results suggest that the expression level of TPH2 affects emotional and cognitive function.

The prevalence of AD in women is higher than that in men, possibly due to the longevity, the education, and the sex hormone estrogen [82]. Several studies revealed that older AD mice, but not younger AD mice, exhibit gender differences during AD pathogenesis [83]. Young females appear to be protected from Aβ toxicity by estrogen due to the anti-inflammatory properties [84, 85]. In the current study, we mainly used male transgenic mouse models to explore the neural circuit regulation of AD-associated depression and cognitive impairments. This regulation in female mice deserves further investigation to fully understand the effects of this circuit on depressive and cognitive impairments in AD mice.

## Conclusion

In summary, we determined that the abnormal hippocampal synaptic function was attributed to the damage of the DRN^5-HT^-dCA1^CaMKII^ neural circuit and overexcitation of CA1^CaMKII^ neurons, mediated by the impaired 5-HT_1B_R and 5-HT_4_R signal transduction in 5×FAD mice. Activating the DRN^5-HT^-dCA1^CaMKII^ neural circuit or applying 5-HT_1B_R and 5-HT_4_R agonists reversed the excessive activation of dCA1^CaMKII^ neurons and improved hippocampal synaptic function, ameliorating early depressive symptoms and cognitive impairments in 5×FAD mice. This highlights the regulatory DRN^5-HT^ neurons as a potential therapeutic target for AD. Early prevention and treatment of AD-related depression may help prevent or delay the cognitive defects of AD.

### Supplementary Information

**Additional file 1: Table S1.** Viruses used in this study. **Table S2.** Antibodies used in this study. **Figure S1.** Changes of TPH2 protein in DRN and dCA1 of 3-month-old or 6-month-old 5×FAD mice. **Figure S2.** No projection from DRN^5-HT^ to dCA1^GABA^. **Figure S3.** Activation of DRN^5-HT^-dCA1^CaMKII^ neural circuit reversed synaptic damage in 5×FAD mice. **Figure S4.** Detection anxiety-like behavior. **Figure S5.** Electrophysiology verified the sustained expression of ChR2 or DREADD in the corresponding neurons of DRN. **Figure S6.** Abnormal expression of serotonergic receptors in the dCA1 of 5×FAD mice. **Figure S7.** Effects of the GABA_A_R blocker picrotoxin on DRN^5-HT^-dCA1^CaMKII^ synaptic transmission.

## Data Availability

All data provided in this paper are available from the corresponding author upon reasonable request.

## Abbreviations

AD: Alzheimer's disease
Aβ: Amyloid β
NFT: Neurofibrillary tangles
CNS: Central nervous system
DRN: Dorsal raphe nucleus
MRN: Medial raphe nucleus
dCA1: Dorsal CA1
GPCR: G-protein-coupled receptors
5-HT_1B_R: 5-HT_1B_ receptors
5-HT_4_R: 5-HT_4_ receptors
CTB: Cholera toxin-B
CNO: Clozapine N-oxide
TST: Tail-suspension test
FST: Forced swimming test
SPT: Sucrose preference test
MWM: Morris Water Maze
sEPSCs: Spontaneous excitatory postsynaptic currents
sIPSCs: Spontaneous inhibitory postsynaptic currents
TPH2: Tryptophan hydroxylase 2
LTP: Long-term potentiation
5-HTRs: 5-HT receptors
PTX: Picrotoxin
VTA/SN: Ventral tegmental area/substantia nigra

## References

[1] Wooten KG, McGuire LC, Olivari BS, Jackson EMJ, Croft JB. Racial and ethnic differences in subjective cognitive decline - United States, 2015–2020. MMWR Morb Mortal Wkly Rep. 2023;72(10):249–55. 36893045 10.15585/mmwr.mm7210a1 PMC10010752

[2] De Lucia N, Carbone G, Muzii B, Ferrara N, Rengo G, Maldonato NM, et al. Neuropsychiatric symptoms and their neural correlates in individuals with mild cognitive impairment. Int Psychogeriatr. 2023;35(11):623–32. 36714990 10.1017/S104161022200117X

[3] Graff-Radford J, Yong KXX, Apostolova LG, Bouwman FH, Carrillo M, Dickerson BC, et al. New insights into atypical Alzheimer’s disease in the era of biomarkers. Lancet Neurol. 2021;20(3):222–34. 33609479 10.1016/S1474-4422(20)30440-3 PMC8056394

[4] Soria Lopez JA, González HM, Léger GC. Alzheimer’s disease. Handb Clin Neurol. 2019;167:231–55. 31753135 10.1016/B978-0-12-804766-8.00013-3

[5] Mittag M, Mediavilla L, Remy S, Cuntz H, Jedlicka P. Modelling the contributions to hyperexcitability in a mouse model of Alzheimer’ disease. J Physiol. 2023;601(15):3403–37. 36734280 10.1113/JP283401

[6] Tzioras M, McGeachan RI, Durrant CS, Spires-Jones TL. Synaptic degeneration in Alzheimer disease. Nat Rev Neurol. 2023;19(1):19–38. 36513730 10.1038/s41582-022-00749-z

[7] Leng F, Hinz R, Gentleman S, Hampshire A, Dani M, Brooks DJ, et al. Neuroinflammation is independently associated with brain network dysfunction in Alzheimer’s disease. Mol Psychiatry. 2023;28(3):1303–11. 36474000 10.1038/s41380-022-01878-z PMC10005956

[8] Lai MK, Tsang SW, Alder JT, Keene J, Hope T, Esiri MM, et al. Loss of serotonin 5-HT2A receptors in the postmortem temporal cortex correlates with rate of cognitive decline in Alzheimer’s disease. Psychopharmacology. 2005;179(3):673–7. 15551121 10.1007/s00213-004-2077-2

[9] Lai MKP, Tsang SWY, Francis PT, Keene J, Hope T, Esiri MM, et al. Postmortem serotoninergic correlates of cognitive decline in Alzheimer’s disease. NeuroReport. 2002;13(9):1175–8. 12151764 10.1097/00001756-200207020-00021

[10] Rodríguez JJ, Noristani HN, Verkhratsky A. The serotonergic system in ageing and Alzheimer’s disease. Prog Neurobiol. 2012;99(1):15–41. 22766041 10.1016/j.pneurobio.2012.06.010

[11] Švob Štrac D, Pivac N, Mück-Šeler D. The serotonergic system and cognitive function. Transl Neurosci. 2016;7(1):35–49. 28123820 10.1515/tnsci-2016-0007 PMC5017596

[12] Yamamoto T, Hirano A. Nucleus raphe dorsalis in Alzheimer’s disease: neurofibrillary tangles and loss of large neurons. Ann Neurol. 1985;17(6):573–7. 4026228 10.1002/ana.410170608

[13] Mowla A, Mosavinasab M, Haghshenas H, Borhani HA. Does serotonin augmentation have any effect on cognition and activities of daily living in Alzheimer’s dementia? A double-blind, placebo-controlled clinical trial. J Clin Psychopharmacol. 2007;27(5):484–7. 17873681 10.1097/jcp.0b013e31814b98c1

[14] Abela AR, Browne CJ, Sargin D, Prevot TD, Ji XD, Li Z, et al. Median raphe serotonin neurons promote anxiety-like behavior via inputs to the dorsal hippocampus. Neuropharmacology. 2020;168:107985. 32035145 10.1016/j.neuropharm.2020.107985

[15] Berger M, Gray JA, Roth BL. The expanded biology of serotonin. Annu Rev Med. 2009;60:355–66. 19630576 10.1146/annurev.med.60.042307.110802 PMC5864293

[16] Tiger M, Varnäs K, Okubo Y, Lundberg J. The 5-HT1B receptor - a potential target for antidepressant treatment. Psychopharmacology. 2018;235(5):1317–34. 29546551 10.1007/s00213-018-4872-1 PMC5919989

[17] Adham N, Romanienko P, Hartig P, Weinshank RL, Branchek T. The rat 5-hydroxytryptamine1B receptor is the species homologue of the human 5-hydroxytryptamine1D beta receptor. Mol Pharmacol. 1992;41(1):1–7. 1732716

[18] Bockaert J, Claeysen S, Compan V, Dumuis A. 5-HT(4) receptors: history, molecular pharmacology and brain functions. Neuropharmacology. 2008;55(6):922–31. 18603269 10.1016/j.neuropharm.2008.05.013

[19] Hannon J, Hoyer D. Molecular biology of 5-HT receptors. Behav Brain Res. 2008;195(1):198–213. 18571247 10.1016/j.bbr.2008.03.020

[20] Fisher JR, Wallace CE, Tripoli DL, Sheline YI, Cirrito JR. Redundant Gs-coupled serotonin receptors regulate amyloid-β metabolism in vivo. Mol Neurodegeneration. 2016;11(1):45. 10.1186/s13024-016-0112-5 PMC4912779 27315796

[21] Montero-Crespo M, Domínguez-Álvaro M, Alonso-Nanclares L, DeFelipe J, Blazquez-Llorca L. Three-dimensional analysis of synaptic organization in the hippocampal CA1 field in Alzheimer’s disease. Brain. 2021;144(2):553–73. 33324984 10.1093/brain/awaa406 PMC8240746

[22] Voigt MM, Laurie DJ, Seeburg PH, Bach A. Molecular cloning and characterization of a rat brain cDNA encoding a 5-hydroxytryptamine1B receptor. EMBO J. 1991;10(13):4017–23. 1836757 10.1002/j.1460-2075.1991.tb04977.x PMC453149

[23] Boeijinga PH, Boddeke HW. Activation of 5-HT1B receptors suppresses low but not high frequency synaptic transmission in the rat subicular cortex in vitro. Brain Res. 1996;721(1–2):59–65. 8793084 10.1016/0006-8993(96)00149-7

[24] Teixeira CM, Rosen ZB, Suri D, Sun Q, Hersh M, Sargin D, et al. Hippocampal 5-HT input regulates memory formation and schaffer collateral excitation. Neuron. 2018;98(5):992–1004. 29754752 10.1016/j.neuron.2018.04.030 PMC6383566

[25] Reynolds GP, Mason SL, Meldrum A, De Keczer S, Parnes H, Eglen RM, et al. 5-Hydroxytryptamine (5-HT)4 receptors in post mortem human brain tissue: distribution, pharmacology and effects of neurodegenerative diseases. Br J Pharmacol. 1995;114(5):993–8. 7780656 10.1111/j.1476-5381.1995.tb13303.x PMC1510307

[26] Restivo L, Roman F, Dumuis A, Bockaert J, Marchetti E, Ammassari-Teule M. The promnesic effect of G-protein-coupled 5-HT4 receptors activation is mediated by a potentiation of learning-induced spine growth in the mouse hippocampus. Neuropsychopharmacology. 2008;33(10):2427–34. 18075492 10.1038/sj.npp.1301644

[27] Marchetti E, Dumuis A, Bockaert J, Soumireu-Mourat B, Roman FS. Differential modulation of the 5-HT(4) receptor agonists and antagonist on rat learning and memory. Neuropharmacology. 2000;39(11):2017–27. 10963745 10.1016/S0028-3908(00)00038-1

[28] Zingg B, Chou XL, Zhang ZG, Mesik L, Liang F, Tao HW, et al. AAV-mediated anterograde transsynaptic tagging: mapping corticocollicular input-defined neural pathways for defense behaviors. Neuron. 2017;93(1):33–47. 27989459 10.1016/j.neuron.2016.11.045 PMC5538794

[29] Wu X, Morishita W, Beier KT, Heifets BD, Malenka RC. 5-HT modulation of a medial septal circuit tunes social memory stability. Nature. 2021;599(7883):96–101. 34616037 10.1038/s41586-021-03956-8 PMC9348902

[30] Leng L, Zhuang K, Liu Z, Huang C, Gao Y, Chen G, et al. Menin deficiency leads to depressive-like behaviors in mice by modulating astrocyte-mediated neuroinflammation. Neuron. 2018;100(3):551–63. 30220511 10.1016/j.neuron.2018.08.031

[31] Page CE, Coutellier L. Prefrontal excitatory/inhibitory balance in stress and emotional disorders: evidence for over-inhibition. Neurosci Biobehav Rev. 2019;105:39–51. 31377218 10.1016/j.neubiorev.2019.07.024

[32] Kjaerby C, Athilingam J, Robinson SE, Iafrati J, Sohal VS. Serotonin 1B receptors regulate prefrontal function by gating callosal and hippocampal inputs. Cell Rep. 2016;17(11):2882–90. 27974203 10.1016/j.celrep.2016.11.036 PMC5268074

[33] Gadgaard C, Jensen AA. Functional characterization of 5-HT1A and 5-HT1B serotonin receptor signaling through G-protein-activated inwardly rectifying K+ channels in a fluorescence-based membrane potential assay. Biochem Pharmacol. 2020;175:113870. 32088264 10.1016/j.bcp.2020.113870

[34] Lanthier C, Dallemagne P, Lecoutey C, Claeysen S, Rochais C. Therapeutic modulators of the serotonin 5-HT4 receptor: a patent review (2014-present). Expert Opin Ther Pat. 2020;30(7):495–508. 32400221 10.1080/13543776.2020.1767587

[35] Castro-Alvarez A, Chávez-Ángel E, Nelson R. Understanding the molecular basis of 5-HT4 receptor partial agonists through 3D-QSAR studies. Int J Mol Sci. 2021;22(7):3602. 33808456 10.3390/ijms22073602 PMC8036435

[36] Toublet F-X, Lecoutey C, Lalut J, Hatat B, Davis A, Since M, et al. Inhibiting acetylcholinesterase to activate pleiotropic prodrugs with therapeutic interest in Alzheimer’s disease. Molecules. 2019;24(15):2786. 31370232 10.3390/molecules24152786 PMC6696315

[37] Yang Y, Zhang L, Yu J, Ma Z, Li M, Wang J, et al. A novel 5-HT1B receptor agonist of herbal compounds and one of the therapeutic uses for Alzheimer’s disease. Front Pharmacol. 2021;12:735876. 34552493 10.3389/fphar.2021.735876 PMC8450432

[38] Nirogi R, Mohammed AR, Shinde AK, Gagginapally SR, Kancharla DM, Ravella SR, et al. Discovery and Preclinical Characterization of Usmarapride (SUVN-D4010): a potent, selective 5-HT4 receptor partial agonist for the treatment of cognitive deficits associated with Alzheimer’s disease. J Med Chem. 2021;64(15):10641–65. 34251799 10.1021/acs.jmedchem.1c00703

[39] Harvey M, Shink E, Tremblay M, Gagné B, Raymond C, Labbé M, et al. Support for the involvement of TPH2 gene in affective disorders. Mol Psychiatry. 2004;9(11):980–1. 15263906 10.1038/sj.mp.4001557

[40] Deng Q, Zhang SQ, Yang PF, Dong WT, Wang JL, Chen JG, et al. A thalamic circuit facilitates stress susceptibility via melanocortin 4 receptor-mediated activation of nucleus accumbens shell. CNS Neurosci Ther. 2023;29(2):646–58. 36510669 10.1111/cns.14046 PMC9873525

[41] Deng Y, Zhou M, Wang J, Yao J, Yu J, Liu W, et al. Involvement of the microbiota-gut-brain axis in chronic restraint stress: disturbances of the kynurenine metabolic pathway in both the gut and brain. Gut Microbes. 2021;13(1):1–16. 33535879 10.1080/19490976.2020.1869501 PMC7872056

[42] Wang B, Shi H, Ren L, Miao Z, Wan B, Yang H, et al. Ahi1 regulates serotonin production by the GR/ERβ/TPH2 pathway involving sexual differences in depressive behaviors. Cell Commun Signal. 2022;20(1):74. 35643536 10.1186/s12964-022-00894-4 PMC9148486

[43] López-Terrones E, Paz V, Campa L, Conde-Berriozabal S, Masana M, Artigas F, et al. Differential modulation of dorsal raphe serotonergic activity in rat brain by the infralimbic and prelimbic cortices. Int J Mol Sci. 2023;24(5):4891. 36902322 10.3390/ijms24054891 PMC10003771

[44] Salvan P, Fonseca M, Winkler AM, Beauchamp A, Lerch JP, Johansen-Berg H. Serotonin regulation of behavior via large-scale neuromodulation of serotonin receptor networks. Nat Neurosci. 2023;26(1):53–63. 36522497 10.1038/s41593-022-01213-3 PMC9829536

[45] Geiller T, Priestley JB, Losonczy A. A local circuit-basis for spatial navigation and memory processes in hippocampal area CA1. Curr Opin Neurobiol. 2023;79:102701. 36878147 10.1016/j.conb.2023.102701 PMC10020891

[46] Steinbusch HWM, Dolatkhah MA, Hopkins DA. Anatomical and neurochemical organization of the serotonergic system in the mammalian brain and in particular the involvement of the dorsal raphe nucleus in relation to neurological diseases. Prog Brain Res. 2021;261:41–81. 33785137 10.1016/bs.pbr.2021.02.003

[47] Ren J, Friedmann D, Xiong J, Liu CD, Ferguson BR, Weerakkody T, et al. Anatomically defined and functionally distinct dorsal raphe serotonin sub-systems. Cell. 2018;175(2):472–87. 30146164 10.1016/j.cell.2018.07.043 PMC6173627

[48] Busche MA, Chen X, Henning HA, Reichwald J, Staufenbiel M, Sakmann B, et al. Critical role of soluble amyloid-β for early hippocampal hyperactivity in a mouse model of Alzheimer’s disease. Proc Natl Acad Sci U S A. 2012;109(22):8740–5. 22592800 10.1073/pnas.1206171109 PMC3365221

[49] Wang J, Mei Y, Zhang X, Wei X, Zhang Y, Wang D, et al. Aberrant serotonergic signaling contributes to the hyperexcitability of CA1 pyramidal neurons in a mouse model of Alzheimer’s disease. Cell Rep. 2023;42(3):112152. 36821438 10.1016/j.celrep.2023.112152

[50] Noristani HN, Meadows RS, Olabarria M, Verkhratsky A, Rodríguez JJ. Increased hippocampal CA1 density of serotonergic terminals in a triple transgenic mouse model of Alzheimer’s disease: an ultrastructural study. Cell Death Dis. 2011;2(9):e210. 21918544 10.1038/cddis.2011.79 PMC3186898

[51] Garcia-Alloza M, Hirst WD, Chen CPLH, Lasheras B, Francis PT, Ramírez MJ. Differential involvement of 5-HT(1B/1D) and 5-HT6 receptors in cognitive and non-cognitive symptoms in Alzheimer’s disease. Neuropsychopharmacology. 2004;29(2):410–6. 14571255 10.1038/sj.npp.1300330

[52] Cho S, Hu Y. Activation of 5-HT4 receptors inhibits secretion of beta-amyloid peptides and increases neuronal survival. Exp Neurol. 2007;203(1):274–8. 16978609 10.1016/j.expneurol.2006.07.021

[53] Giannoni P, Gaven F, de Bundel D, Baranger K, Marchetti-Gauthier E, Roman FS, et al. Early administration of RS 67333, a specific 5-HT4 receptor agonist, prevents amyloidogenesis and behavioral deficits in the 5×FAD mouse model of Alzheimer’s disease. Front Aging Neurosci. 2013;5:96. 24399967 10.3389/fnagi.2013.00096 PMC3871961

[54] Donovan NJ, Hsu DC, Dagley AS, Schultz AP, Amariglio RE, Mormino EC, et al. Depressive symptoms and biomarkers of Alzheimer’s disease in cognitively normal older adults. J Alzheimers Dis. 2015;46(1):63–73. 25697700 10.3233/JAD-142940 PMC4544638

[55] Lokuge S, Frey BN, Foster JA, Soares CN, Steiner M. Depression in women: windows of vulnerability and new insights into the link between estrogen and serotonin. J Clin Psychiatry. 2011;72(11):e1563–9. 22127200 10.4088/JCP.11com07089

[56] Cools R, Roberts AC, Robbins TW. Serotoninergic regulation of emotional and behavioural control processes. Trends Cogn Sci. 2008;12(1):31–40. 18069045 10.1016/j.tics.2007.10.011

[57] Ramirez MJ, Lai MKP, Tordera RM, Francis PT. Serotonergic therapies for cognitive symptoms in Alzheimer’s disease: rationale and current status. Drugs. 2014;74(7):729–36. 24802806 10.1007/s40265-014-0217-5

[58] Schmitz D, Gloveli T, Empson RM, Draguhn A, Heinemann U. Serotonin reduces synaptic excitation in the superficial medial entorhinal cortex of the rat via a presynaptic mechanism. J Physiol. 1998;508(Pt 1):119–29. 9490827 10.1111/j.1469-7793.1998.119br.x PMC2230865

[59] Adhikari A, Topiwala MA, Gordon JA. Single units in the medial prefrontal cortex with anxiety-related firing patterns are preferentially influenced by ventral hippocampal activity. Neuron. 2011;71(5):898–910. 21903082 10.1016/j.neuron.2011.07.027 PMC3201792

[60] Muzerelle A, Scotto-Lomassese S, Bernard JF, Soiza-Reilly M, Gaspar P. Conditional anterograde tracing reveals distinct targeting of individual serotonin cell groups (B5–B9) to the forebrain and brainstem. Brain Struct Funct. 2016;221(1):535–61. 25403254 10.1007/s00429-014-0924-4 PMC4750555

[61] Ohmura Y, Tsutsui-Kimura I, Sasamori H, Nebuka M, Nishitani N, Tanaka KF, et al. Different roles of distinct serotonergic pathways in anxiety-like behavior, antidepressant-like, and anti-impulsive effects. Neuropharmacology. 2020;167:107703. 31299228 10.1016/j.neuropharm.2019.107703

[62] Meltzer CC, Smith G, DeKosky ST, Pollock BG, Mathis CA, Moore RY, et al. Serotonin in aging, late-life depression, and Alzheimer’s disease: the emerging role of functional imaging. Neuropsychopharmacology. 1998;18(6):407–30. 9571651 10.1016/S0893-133X(97)00194-2

[63] Silk E, Diwan M, Rabelo T, Katzman H, Campos ACP, Gouveia FV, et al. Serotonin 5-HT1B receptors mediate the antidepressant- and anxiolytic-like effects of ventromedial prefrontal cortex deep brain stimulation in a mouse model of social defeat. Psychopharmacology. 2022;239(12):3875–92. 36282287 10.1007/s00213-022-06259-6

[64] Li X, Liang S, Li Z, Li S, Xia M, Verkhratsky A, et al. Leptin increases expression of 5-HT2B receptors in astrocytes thus enhancing action of fluoxetine on the depressive behavior induced by sleep deprivation. Front Psychiatry. 2018;9:734. 30666218 10.3389/fpsyt.2018.00734 PMC6330762

[65] King MV, Marsden CA, Fone KCF. A role for the 5-HT(1A), 5-HT4 and 5-HT6 receptors in learning and memory. Trends Pharmacol Sci. 2008;29(9):482–92. 19086256 10.1016/j.tips.2008.07.001

[66] Naumenko VS, Popova NK, Lacivita E, Leopoldo M, Ponimaskin EG. Interplay between serotonin 5-HT1A and 5-HT7 receptors in depressive disorders. CNS Neurosci Ther. 2014;20(7):582–90. 24935787 10.1111/cns.12247 PMC6493079

[67] Zhang QJ, Li LB, Niu XL, Liu J, Gui ZH, Feng JJ, et al. The pyramidal neurons in the medial prefrontal cortex show decreased response to 5-hydroxytryptamine-3 receptor stimulation in a rodent model of Parkinson’s disease. Brain Res. 2011;1384:69–79. 21291871 10.1016/j.brainres.2011.01.086

[68] Schmitz D, Empson RM, Heinemann U. Serotonin and 8-OH-DPAT reduce excitatory transmission in rat hippocampal area CA1 via reduction in presumed presynaptic Ca2+ entry. Brain Res. 1995;701(1–2):249–54. 8925288 10.1016/0006-8993(95)01005-5

[69] Torres-Escalante JL, Barral JA, Ibarra-Villa MD, Pérez-Burgos A, Góngora-Alfaro JL, Pineda JC. 5-HT1A, 5-HT2, and GABAB receptors interact to modulate neurotransmitter release probability in layer 2/3 somatosensory rat cortex as evaluated by the paired pulse protocol. J Neurosci Res. 2004;78(2):268–78. 15378508 10.1002/jnr.20247

[70] Mlinar B, Falsini C, Corradetti R. Pharmacological characterization of 5-HT(1B) receptor-mediated inhibition of local excitatory synaptic transmission in the CA1 region of rat hippocampus. Br J Pharmacol. 2003;138(1):71–80. 12522075 10.1038/sj.bjp.0705026 PMC1573652

[71] Pickard GE, Smith BN, Belenky M, Rea MA, Dudek FE, Sollars PJ. 5-HT1B receptor-mediated presynaptic inhibition of retinal input to the suprachiasmatic nucleus. J Neurosci. 1999;19(10):4034–45. 10234032 10.1523/JNEUROSCI.19-10-04034.1999 PMC6782735

[72] Singer JH, Bellingham MC, Berger AJ. Presynaptic inhibition of glutamatergic synaptic transmission to rat motoneurons by serotonin. J Neurophysiol. 1996;76(2):799–807. 8871200 10.1152/jn.1996.76.2.799

[73] Dawson LA, Nguyen HQ, Li P. The 5-HT(6) receptor antagonist SB-271046 selectively enhances excitatory neurotransmission in the rat frontal cortex and hippocampus. Neuropsychopharmacology. 2001;25(5):662–8. 11682249 10.1016/S0893-133X(01)00265-2

[74] Katsurabayashi S, Kubota H, Tokutomi N, Akaike N. A distinct distribution of functional presynaptic 5-HT receptor subtypes on GABAergic nerve terminals projecting to single hippocampal CA1 pyramidal neurons. Neuropharmacology. 2003;44(8):1022–30. 12763095 10.1016/S0028-3908(03)00103-5

[75] Kishimoto K, Koyama S, Akaike N. Synergistic mu-opioid and 5-HT1A presynaptic inhibition of GABA release in rat periaqueductal gray neurons. Neuropharmacology. 2001;41(5):529–38. 11587707 10.1016/S0028-3908(01)00100-9

[76] Koyama S, Matsumoto N, Murakami N, Kubo C, Nabekura J, Akaike N. Role of presynaptic 5-HT1A and 5-HT3 receptors in modulation of synaptic GABA transmission in dissociated rat basolateral amygdala neurons. Life Sci. 2002;72(4–5):375–87. 12467878 10.1016/S0024-3205(02)02280-4

[77] Jacobs BL, Azmitia EC. Structure and function of the brain serotonin system. Physiol Rev. 1992;72(1):165–229. 1731370 10.1152/physrev.1992.72.1.165

[78] Matsuoka T, Hasuo H, Akasu T. 5-Hydroxytryptamine 1B receptors mediate presynaptic inhibition of monosynaptic IPSC in the rat dorsolateral septal nucleus. Neurosci Res. 2004;48(3):229–38. 15154669 10.1016/j.neures.2003.11.004

[79] Willette AA, Pappas C, Hoth N, Wang Q, Klinedinst B, Willette SA, et al. Inflammation, negative affect, and amyloid burden in Alzheimer’s disease: Insights from the kynurenine pathway. Brain Behav Immun. 2021;95:216–25. 33775832 10.1016/j.bbi.2021.03.019 PMC8187283

[80] Oxenkrug GF. Genetic and hormonal regulation of tryptophan kynurenine metabolism: implications for vascular cognitive impairment, major depressive disorder, and aging. Ann N Y Acad Sci. 2007;1122:35–49. 18077563 10.1196/annals.1403.003

[81] Komleva PD, Alhalabi G, Izyurov AE, Khotskin NV, Kulikov AV. Effects of the combination of the C1473G mutation in the Tph2 gene and lethal yellow mutations in the Raly-Agouti locus on behavior, brain 5-HT and melanocortin systems in mice. Biomolecules. 2023;13(6):963. 37371543 10.3390/biom13060963 PMC10295981

[82] 2022 Alzheimer’s disease facts and figures. Alzheimers Dement. 2022;18(4):700–789. 10.1002/alz.12638. 10.1002/alz.12638 35289055

[83] Stevens LM, Brown RE. Reference and working memory deficits in the 3xTg-AD mouse between 2 and 15-months of age: a cross-sectional study. Behav Brain Res. 2015;278:496–505. 25446812 10.1016/j.bbr.2014.10.033

[84] Viña J, Lloret A. Why women have more Alzheimer’s disease than men: gender and mitochondrial toxicity of amyloid-beta peptide. J Alzheimers Dis. 2010;20(Suppl 2):S527–33. 20442496 10.3233/JAD-2010-100501

[85] Yang JT, Wang ZJ, Cai HY, Yuan L, Hu MM, Wu MN, et al. Sex differences in neuropathology and cognitive behavior in APP/PS1/tau triple-transgenic mouse model of Alzheimer’s disease. Neurosci Bull. 2018;34(5):736–46. 30099679 10.1007/s12264-018-0268-9 PMC6129237

