# Supplementary material for: Dorsal raphe nucleus–hippocampus serotonergic circuit underlies the depressive and cognitive impairments in 5×FAD male mice
Source: Transl Neurodegener. 2024 Jul 24;13:34. doi: 10.1186/s40035-024-00425-w (PMC11267773; doi:10.1186/s40035-024-00425-w)
Supplement: Supplementary file 1 — Additional file 1: Table S1. Viruses used in this study. Table S2. Antibodies used in this study. Figure S1. Changes of TPH2 protein in DRN and dCA1 of 3-month-old or 6-month-old 5×FAD mice. Figure S2. No projection from DRN5-HT to dCA1GABA. Figure S3. Activation of DRN5-HT-dCA1CaMKII neural circuit reversed synaptic damage in 5×FAD mice. Figure S4. Detection anxiety-like behavior. Figure S5. Electrophysiology verified the sustained expression of ChR2 or DREADD in the corresponding neurons of DRN. Figure S6. Abnormal expression of serotonergic receptors in the dCA1 of 5×FAD mice. Figure S7. Effects of the GABAAR blocker picrotoxin on DRN5-HT-dCA1CaMKII synaptic transmission. [file 40035_2024_425_MOESM1_ESM.docx]

**Supplemental Information**

**Dorsal raphe nucleus–Hippocampus serotonergic circuit underlies the depressive and cognitive impairments in 5×FAD male mice**

Meiqin Chen^1,2^, Chenlu Wang^3^, Yinan Lin^3^, Yanbing Chen^2^, Wenting Xie^2^, Xiaoting Huang^2^, Fan Zhang^1^, Congrui Fu^1^, Kai Zhuang^2^, Tingting Zou^2^, Dan Can^2^, Huifang Li^2^, Shengxi Wu^4^, Ceng Luo^4^, Jie Zhang^1,2,5,6,*^

1. The Key Laboratory of Neural and Vascular Biology, Ministry of Education, College of Basic Medicine, Hebei Medical University, Shijiazhuang, 050017, China
2. Institute of Neuroscience, College of Medicine, Xiamen University, Xiamen 361102, China
3. Department of Anesthesiology, First Affiliated Hospital of Xiamen University, Xiamen, 361000, China
4. Department of Neurobiology, School of Basic Medicine, Fourth Military Medical University, Xi’an, 710032, China
5. Department of Neurology, Sichuan Provincial People's Hospital, School of Medicine, University of Electronic Science and Technology of China, Chengdu, 610054, China.
6. Institute of Neuroscience, Fujian Medical University, Fuzhou, 350004, China.

*To whom correspondence should be addressed:

Jie Zhang, Phone/Fax: (86)592-2180717; E-mail: [jiezhang@xmu.edu.cn](mailto:jiezhang@xmu.edu.cn); Institute of Neuroscience, College of Medicine, Xiamen University, Xiamen, Fujian 361005, China

**Table S1:** Viruses used in this study.

| **Virus strains** | **Source** | **Identifier** |
| --- | --- | --- |
| rAAV2/9-TPH2-CRE-WPRE-pA | BrainVTA | Cat# PT-0390 |
| rAAV2/R-TPH2-CRE-WPRE-pA | BrainVTA | Cat# PT-0396 |
| rAAV2/1-TPH2-CRE-WPRE-pA | BrainVTA | Cat# PT-0396 |
| rAAV2/9-Ef1α-Dio-hM3D(Gq)-mCherry-WPRE-pA | BrainVTA | Cat# PT-0042 |
| rAAV2/9-Ef1α-Dio-mCherry-WPRE-pA | BrainVTA | Cat# PT-0013 |
| rAAV2/9-Ef1α-Dio-hChR2(H134R)-mCherry-WPRE-pA | BrainVTA | Cat# PT-0002 |
| CTB-555 | BrainVTA | Cat# CT-02 |
| rAAV2/9-EF1α-Dio-H2B-EGFP-T2A-TVA-WPRE-hGH-pA | BrainVTA | Cat# PT-0021 |
| rAAV2/9-EF1α-Dio-oRVG-WPRE-pA | BrainVTA | Cat# PT-0023 |
| RV-EnVA-ΔG-dsRed | BrainVTA | Cat# R01002 |
| rAAV-TPH2-mCherry-WPRE-hGH | BrainVTA | Cat# PT-2484 |
| rAAV-TPH2-Tph2-P2A-mCherry-WPRE-hGH | BrainVTA | Cat# PT-6576 |

**Table S2:** Antibodies used in this study.

| **Antibodies** | **Source** | **Identifier** |
| --- | --- | --- |
| Polyclonal rabbit anti-c-Fos | Cell signaling technology | Cat#2250S, RRID: AB_2247211 |
| Polyclonal rabbit anti-APP | Sangon | Cat# D260097-0025 |
| Polyclonal rabbit anti-TPH2 | Novus | Cat# NB100-74555, RRID: AB_1049988 |
| Polyclonal mouse anti-GAPDH | Proteintech | Cat# 60004-1-1g, RRID: AB_2107436 |
| Polyclonal rabbit anti-CaMKⅡα | Novus | Cat# NB100-1983, RRID: AB_10001339 |
| Polyclonal rabbit anti-cleaved-caspase-3 | Cell signaling technology | Cat#9661S |


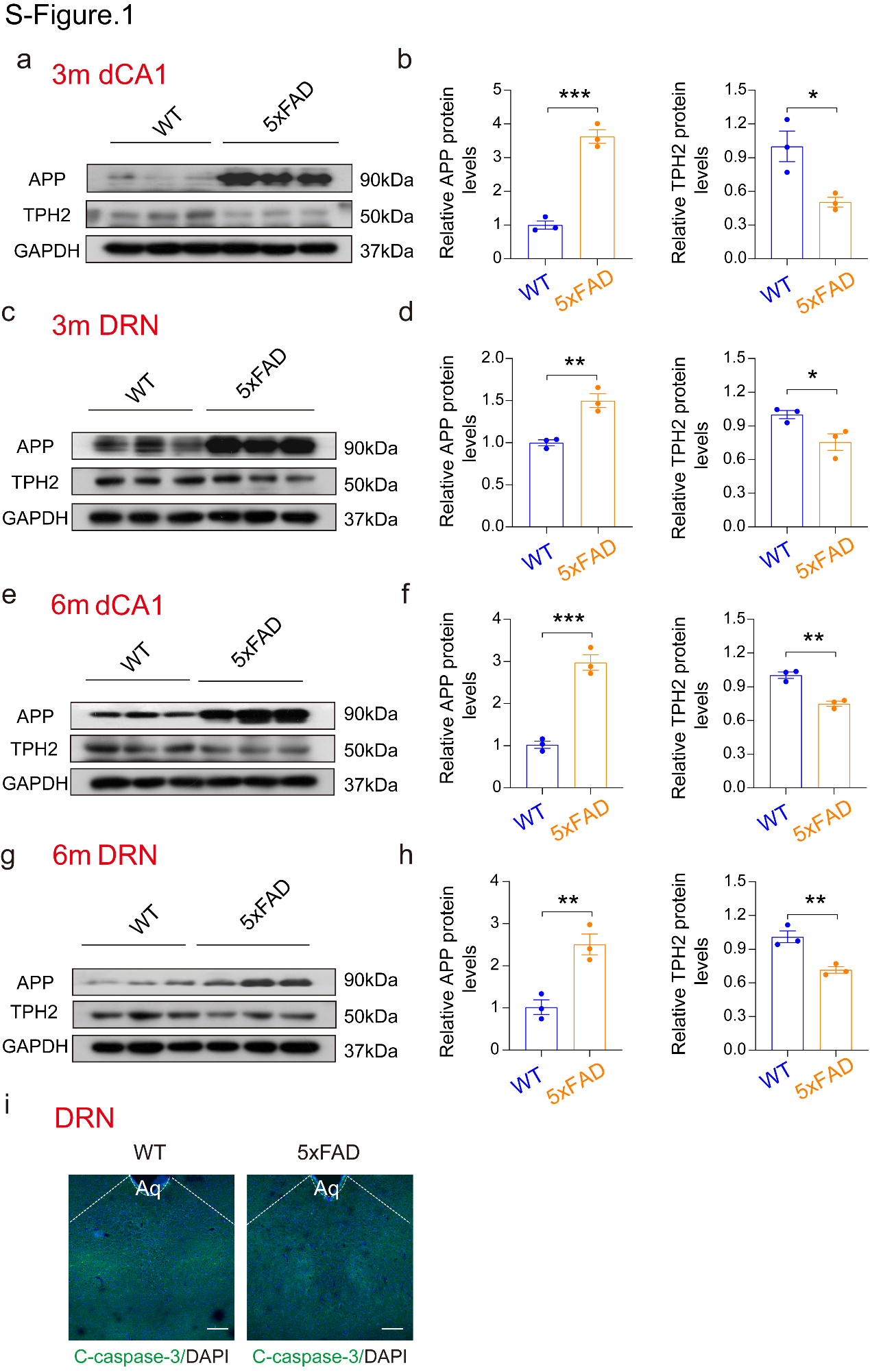


**Figure S1. Changes of TPH2 protein in DRN and dCA1 of 3-month-old or 6-month-old 5×FAD mice.**

(**a**) Representation of APP and TPH2 expression in the dCA1.

(**b**) Quantitative analysis of APP and TPH2 expression in the dCA1. The data are expressed as the mean ± SEM, *n=*3 per group, **P <* 0.05, ***P <* 0.01, ****P <* 0.001, by two-tailed Student’s *t*-test.

(**c**) Representation of APP and TPH2 expression in the DRN.

(**d**) Quantitative analysis of APP and TPH2 expression in the DRN. The data are expressed as the mean ± SEM, *n=*3 per group, **P <* 0.05, ***P <* 0.01, by two-tailed Student’s *t*-test.

(**e**) Representation of APP and TPH2 expression in the dCA1.

(**f**) Quantitative analysis of APP and TPH2 expression in dCA1. The data are expressed as the mean ± SEM, *n=*3 per group **P <* 0.05, ***P <* 0.01, ****P <* 0.01, by two-tailed Student’s *t*-test.

(**g**) Representation of APP and TPH2 expression in the DRN.

(**h**) Quantitative analysis of APP and TPH2 expression in DRN. The data are expressed as the mean ± SEM, *n* = 3 per group **P <* 0.05, ***P <* 0.01, by two-tailed Student’s *t*-test*.*

(**i**) Immunofluorescence staining for cleaved caspase-3 as an apoptotic marker in DRN of WT and 5×FAD mice. C-caspase-3 (green), DAPI (blue). Scale bar, 200μm.


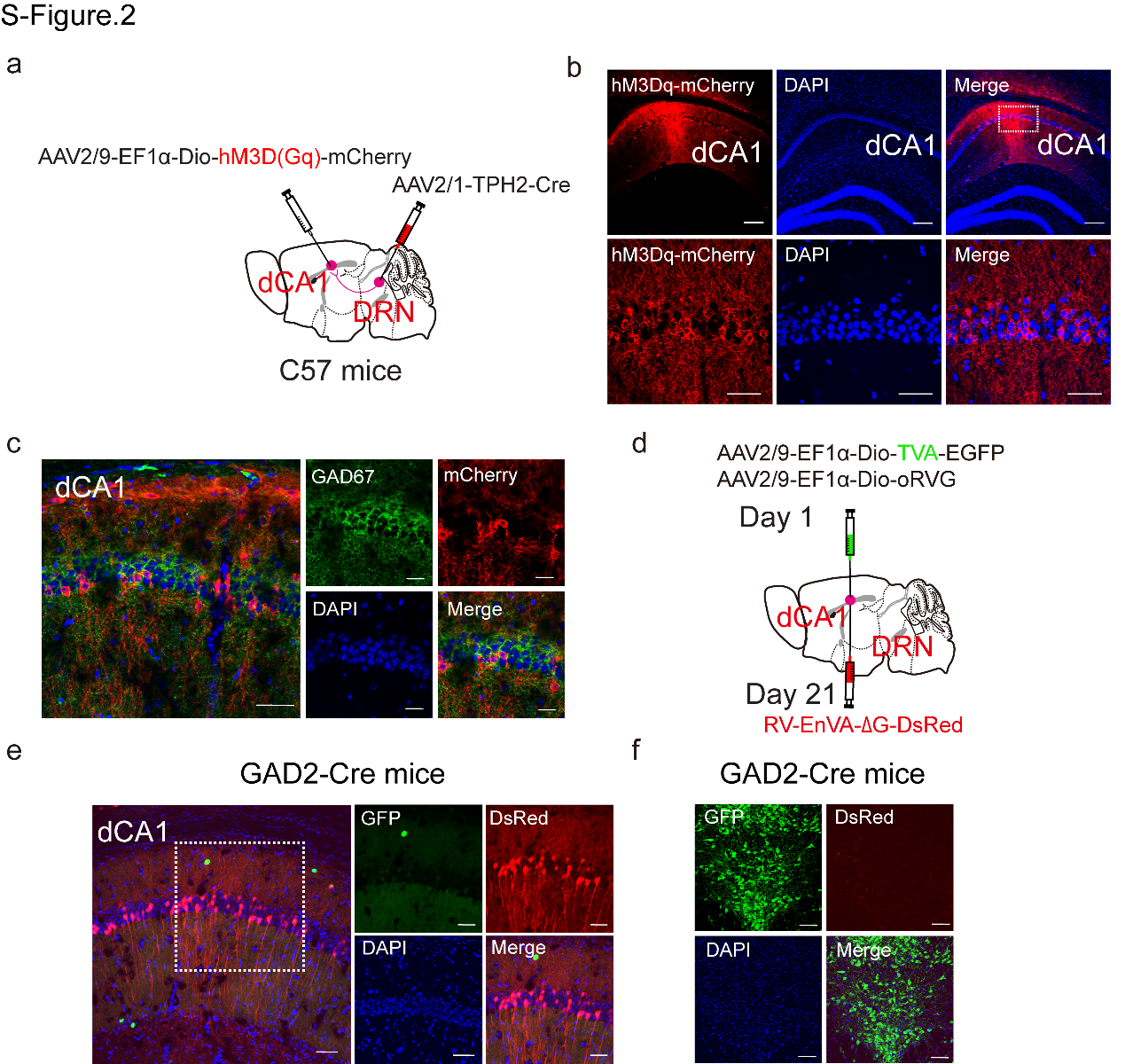


**Figure S2. No projection from DRN^5-HT^ to dCA1^GABA^.**

(**a**) Schematic of anterograde labeled virus tracing strategy.

(**b**) Representation of anterograde labeled virus.

(**c**) The mCherry signals were not co-localized with GAD67 immunofluorescence in the dCA1. mCherry (red), GAD67 (green), and DAPI (blue). Scale bar, 5 μm.

(**d**) Schematic of the Cre-dependent retrograde trans-monosynaptic rabies virus tracing strategy.

(**e**) Representative images of viral expression within the dCA1 of *GAD2-Cre* mice. Scale bars, 100 μm (left) and 50 μm (right).

(**f**) No DsRed signals were identified in the DRN of *GAD2-Cre* mice. Scale bar, 200 µm.


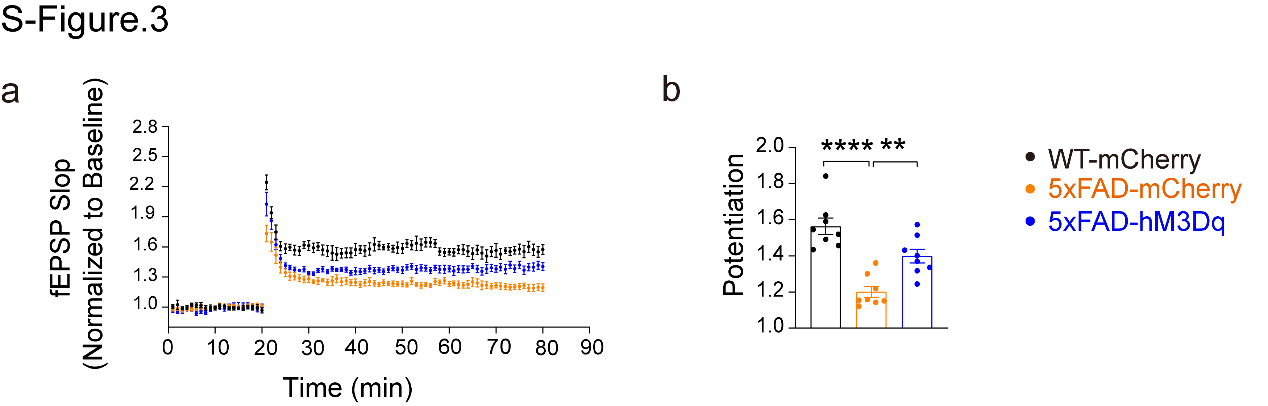


**Figure S3. Activation of the DRN^5-HT^-dCA1^CaMKⅡ^ neural circuit reversed synaptic damage in 5×FAD mice.**

(**a**) LTP was induced by two trains of 100-Hz stimuli in the Schaffer collaterals

(**b**) Significant differences in fEPSP potentiation were determined by comparing fEPSP slopes during the last 10 min of recording after high-frequency stimulation. WT-mCherry = 8 slices from 5 mice, 5×FAD-mCherry = 8 slices from 5 mice, 5×FAD-hM3Dq = 8 slices from 5 mice. The data are expressed as the mean ± SEM, ***P <* 0.01, *****P <* 0.0001, by One-way ANOVA followed by Holm-Sidak pairwise test.


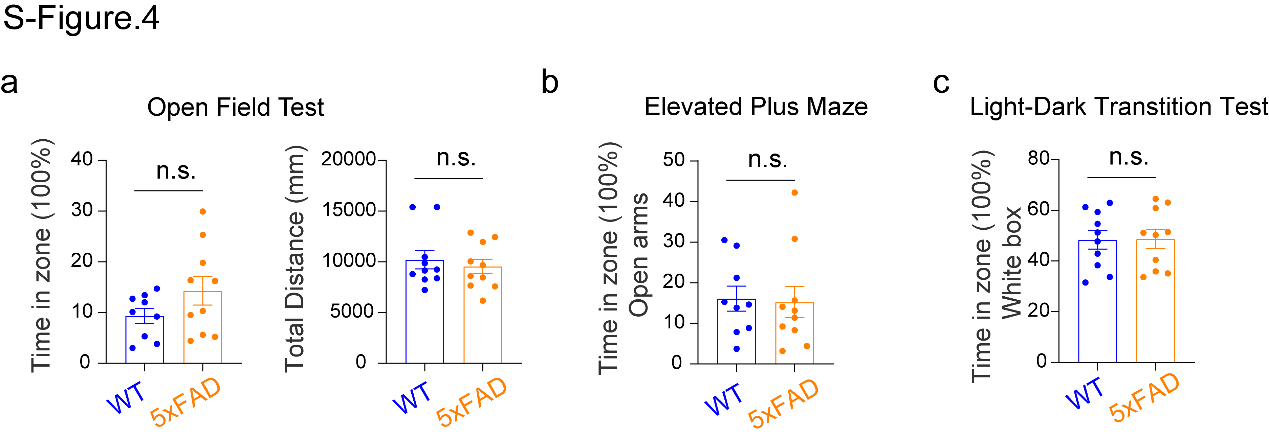


**Figure S4. Detection anxiety-like behavior.**

(**a**) Total distance and center entries in OFT.

(**b**) Duration spent in the open arms in EPM.

(**c**) Duration spent in the white box in LDT.


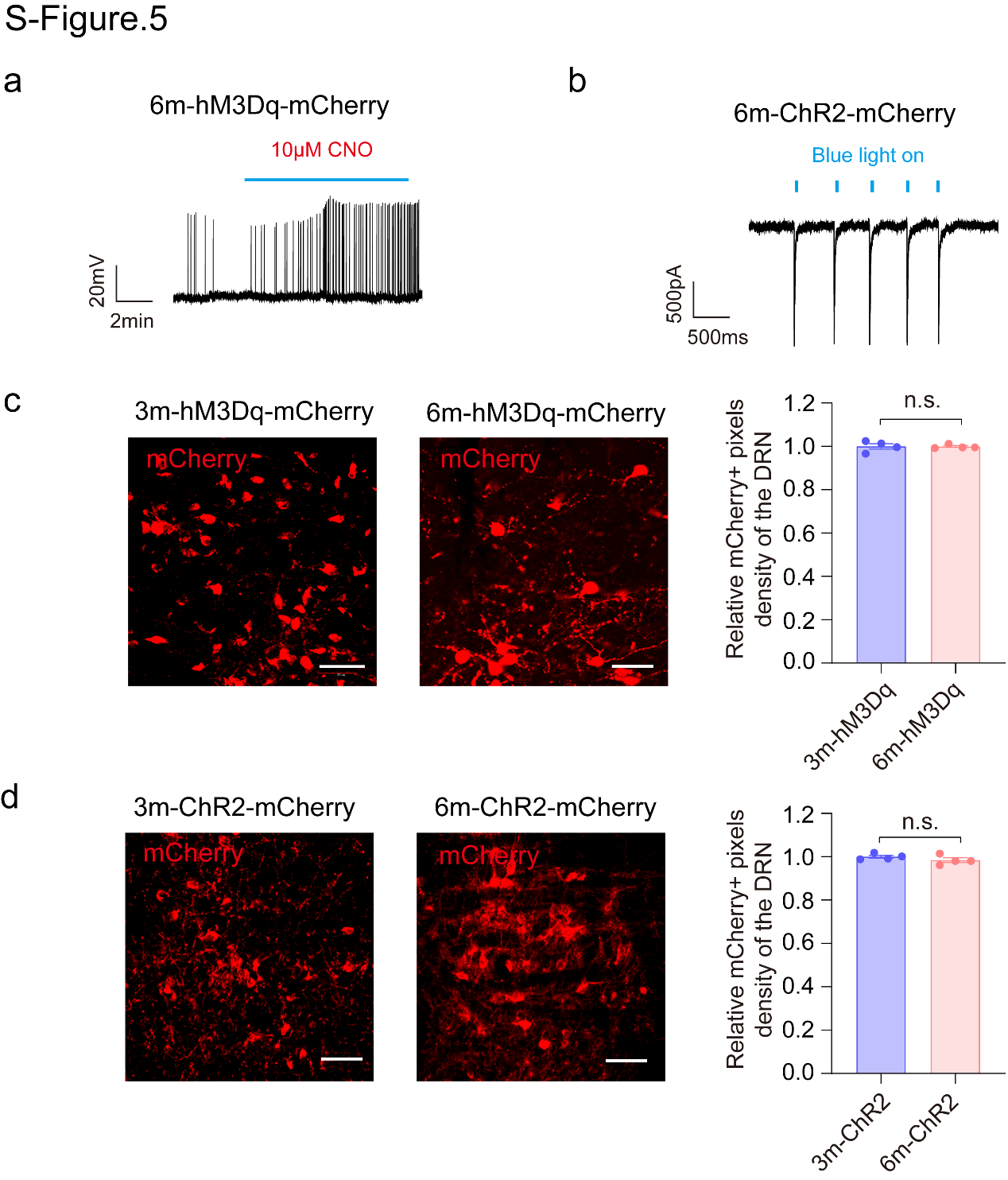


**Figure S5. Electrophysiology verified the sustained expression of ChR2 or DREADD in the corresponding neurons of DRN.**

(**a**) Representative trace of Gq-DREADD neurons in DRN from 6-month-old mice (the virus was expressed for 4 months).

(**b**) Sample traces of action potentials evoked by 473 nm light (blue light) recorded from DRN mCherry+ neurons in acute slices of 6-month-old mice (the virus was expressed for 4 months).

(**c**) Representation and quantification of DREADD expression efficiency in DRNS of 3-month-old and 6-month-old mice. (3m-hM3Dq-mCherry = 4 mice, 6m-hM3Dq-mCherry = 4 mice). The data are expressed as the mean ± SEM, **P* < 0.05, by two-tailed Student’s *t*-test.

(**d**) Representation and quantification of ChR2 expression efficiency in DRN of 3-month-old and 6-month-old mice. (3m-ChR2-mCherry = 4 mice, 6m-ChR2-mCherry = 4 mice). The data are expressed as the mean ± SEM, **P* < 0.05, by two-tailed Student’s *t*-test.


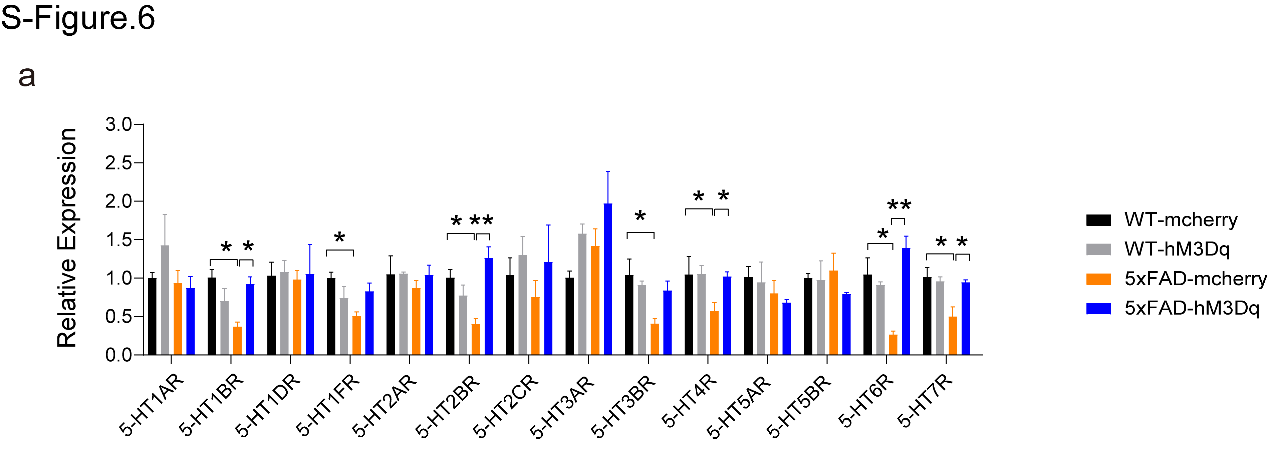


**Figure S6. Abnormal expression of serotonergic receptors in the dCA1 of 5×FAD mice.**

(**a**) Effect of chemogenetic activation of DRN 5-HT neurons in the DRN-CA1 neural circuit on the relative expression of 5-HT receptors from transcriptional analysis. Mean ± SEM, **P <* 0.05, ***P <* 0.01, by One-way ANOVA, followed by Holm-Sidak pairwise test.


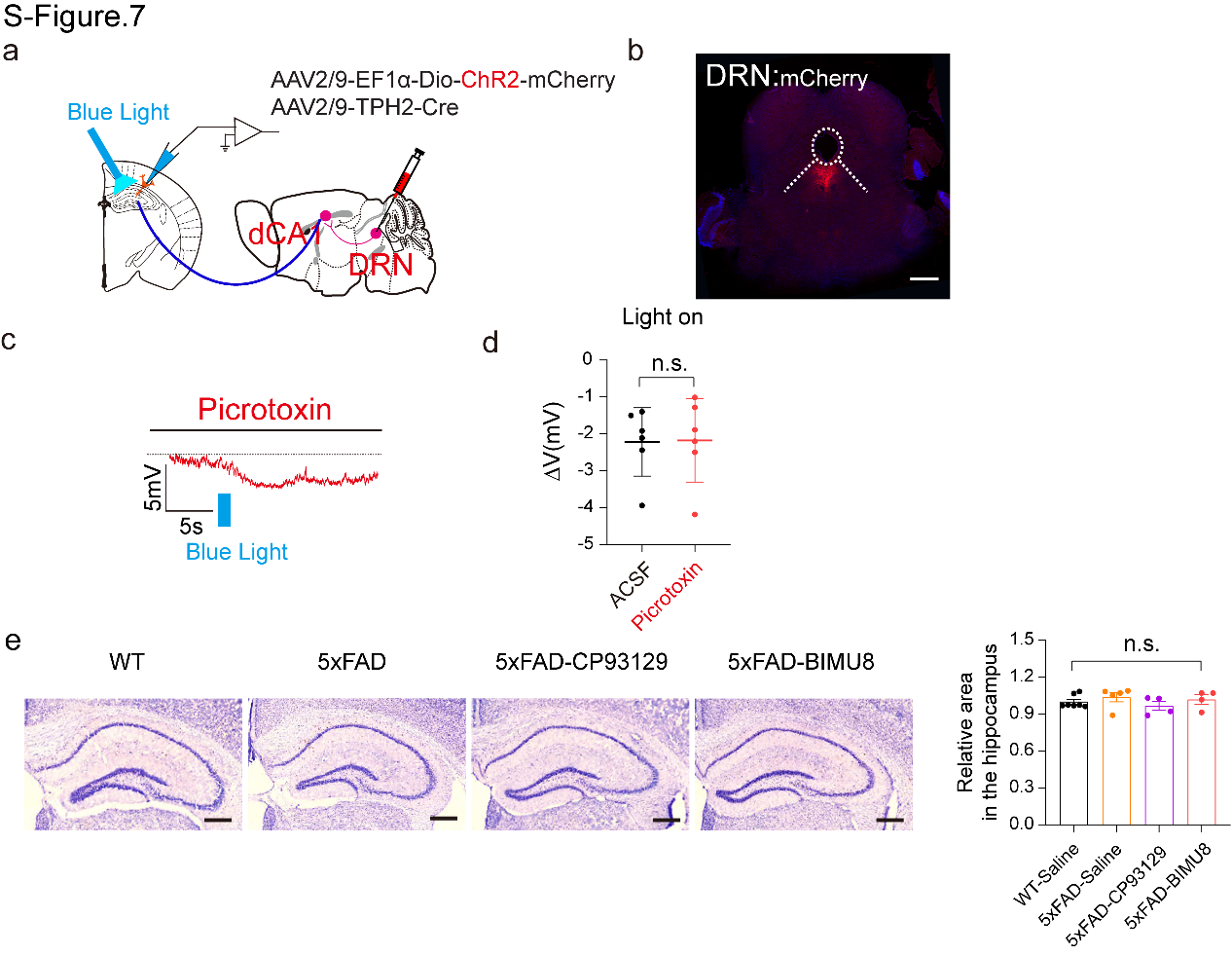


**Figure S7. Effects of the GABA_A_R blocker picrotoxin on DRN^5-HT^-dCA1^CaMKⅡ^ synaptic transmission.**

(**a**) Schematic showing the injection of AAV2/9-Dio-ChR2-mCherry and AAV2/9-TPH2-Cre into the DRN and whole-cell patch recording of dCA1 neurons.

(**b**) A representative image of viral expression within the DRN. Scale bar, 1mm.

(**c**-**d**) A sample trace and summarized data of hyperpolarized potentials of dCA1^CaMKⅡ^ neurons evoked by photostimulation in the presence of picrotoxin. ACSF = 6 cells from 6 mice，Picrotoxin = 6 cells from 6 mice. All data are displayed as mean ± SEM, ns, no significance, by two-tailed Student’s *t*-test.

(**e**) Nissl staining of the hippocampus. Scale bar, 500 μm.
